# Artificial neural networks enable genome-scale simulations of intracellular signaling

**DOI:** 10.1038/s41467-022-30684-y

**Published:** 2022-06-02

**Authors:** Avlant Nilsson, Joshua M. Peters, Nikolaos Meimetis, Bryan Bryson, Douglas A. Lauffenburger

**Affiliations:** 1 Department of Biological Engineering, Massachusetts Institute of Technology, Cambridge, MA 02139 USA; 2 Department of Biology and Biological Engineering, Chalmers University of Technology, Gothenburg, SE 41296 Sweden; 3 Ragon Institute of MGH, MIT, and Harvard, Cambridge, MA 02139 USA

**Keywords:** Computer modelling, Biochemical networks, Cellular signalling networks, Regulatory networks

## Abstract

Mammalian cells adapt their functional state in response to external signals in form of ligands that bind receptors on the cell-surface. Mechanistically, this involves signal-processing through a complex network of molecular interactions that govern transcription factor activity patterns. Computer simulations of the information flow through this network could help predict cellular responses in health and disease. Here we develop a recurrent neural network framework constrained by prior knowledge of the signaling network with ligand-concentrations as input and transcription factor-activity as output. Applied to synthetic data, it predicts unseen test-data (Pearson correlation *r* = 0.98) and the effects of gene knockouts (*r* = 0.8). We stimulate macrophages with 59 different ligands, with and without the addition of lipopolysaccharide, and collect transcriptomics data. The framework predicts this data under cross-validation (*r* = 0.8) and knockout simulations suggest a role for RIPK1 in modulating the lipopolysaccharide response. This work demonstrates the feasibility of genome-scale simulations of intracellular signaling.

## Abstract (web summary)

Many diseases are caused by disruptions to the network of biochemical reactions that allow cells to respond to external signals. Here Nilsson et al develop a method to simulate cellular signaling using artificial neural networks to predict cellular responses and activities of signaling molecules.

## Introduction

The healthy body continuously adapts to the environment by altering the molecular state of its cells. This primarily occurs through binding of multiple types of ligands to receptors at the cell-surface, this acts as signals that are propagated through molecular interactions culminating in activation of transcription factors (TF) and subsequent transcription of genes. Rather than constituting independent paths from receptors to specific genes, signaling is conducted through a complex network with spatial and temporal components^1^. This enables the cell to compute a response to stimulation by multiple ligands^2,3^, e.g., co-stimulation of human macrophages gives rise to a spectrum of cellular activation states^4^. Disruptions to the network can cause disease, e.g., activating mutations in the signaling protein BRAF is present in 40–50% of all melanoma tumors, i.e., skin cancer, and single target treatments are not always sufficient due to cellular adaptations, e.g., tumors often acquire resistance to BRAF-inhibitors^5^. A systems perspective on signaling is required to better understand responses to co-stimulation and predict the effects of drugs. Such an understanding could be obtained through genome-scale computer simulations of signaling that have long been anticipated^6–8^.

By now, many requisites for genome-scale models of signaling are in place. The network topology has been extensively characterized with thousands of biochemical interactions collected in databases^9^ and with visual maps available for many signaling pathways, e.g., through the Kyoto Encyclopedia of Genes and Genomes (KEGG)^10^. Genome wide data can be generated using high-throughput methods, e.g., activities of hundreds of TFs can be statistically inferred from transcriptomics data^11^ and cellular responses to combinations of ligands, can be characterized through co-stimulation experiments^2^. For metabolism, genome-scale simulations are routinely performed using the flux balance analysis (FBA) framework, which predicts intracellular fluxes using steady state assumptions, linear optimization, and data on metabolic exchange rates^12^. It has been used to gain system level insight on a wide range of topics, e.g., the effect of intercellular compartmentalization on the flux of glutamate in cancer^13^ or the influence of metabolic trade-offs on oxygen consumption in muscle cells^14^. However, the linear FBA methodology cannot be applied to signaling, in which nonlinear relationships are typically important to capture and stoichiometric constraints are less straightforward to impose.

Current signaling models are often based on ordinary differential equations (ODE) or logic rules^7,8,15^ and face challenges when expanding to the genome-scale^7,16^. Yet, several of these have been overcome by simplifying assumptions. Explicit enumeration of microstates, which has been successful for individual proteins, is numerically intractable at the genome-scale^17^ due to a combinatorial explosion of states from posttranslational modifications and protein complexes. This is circumvented by models that omit enumeration, e.g., signal flow models^15^, which represent signaling as a signed directed graph with scalar activity values for each signaling molecule. Cellular activity occurs across multiple timescales, e.g., conformational changes of proteins occurs much faster than signaling events, while protein translation from mRNA occurs much slower. The requirement by network-wide models for simulation of long time-courses at high resolution can be overcome using quasi-steady-state approximations^17,18^ that assume that faster processes are instantaneous and slower processes are constant. However, two major limitations remain for reaching the genome-scale using current methods: predefined equations are needed for each molecule, while the exact mechanism is often unknown; and parameter estimation may require problematically long computational times for the largest models despite major advances^19^. Therefore, an alternative framework for modeling signaling is warranted.

Advancements in artificial neural networks (ANN) have enabled large-scale models in many different areas, including drug discovery and genomics^20^. ANNs approximate unknown and highly complex functions through a sequence of linear matrix operations and non-linear transformations. These approximations, sometimes containing millions of parameters, can be rapidly trained from paired samples of input and output data using the backpropagation algorithm^20,21^. While ANNs excel at predictions, their underlying mechanism is often elusive, and therefore more interpretable ANNs based on prior knowledge have been proposed for modeling biological systems^22^. For example, a feed forward neural network (FFNN) with a network topology derived from known signaling interactions has been used to predict cell types from gene expression data^22^. However, FFNN do not allow feedback loops, which are frequent in signaling, and therefore recurrent neural networks (RNN) may be a more suitable architecture for modeling signaling networks. It has previously been shown that a RNN without prior knowledge constraints can recapitulate the output of a small ODE-model of signaling^23^.

Here we construct a framework for rapid parameterization and simulation of intracellular signaling using RNNs hence referred to as LEMBAS, Large-scale knowledge-EMBedded Artificial Signaling-networks. We first construct an activation function suitable for approximating the steady state behavior of different molecular mechanisms. We then introduce a sparse RNN formalism that encodes the topology of a known signaling network. LEMBAS uses ligand concentrations as input to predict TF activities at steady state and we construct a regularization function that ensures that steady state is reached. To test the data requirements for training generalizable models, we generate synthetic data from a reference model with computationally derived parameters. Models trained on modestly sized (400–800 samples) synthetic datasets, accurately predict most randomly generated input-output pairs from the reference model. Additionally, the trained model predicts the effect of simulated gene knock outs (KO). To demonstrate the frameworks applicability to real world data, we generate a transcriptomics dataset for macrophages stimulated with 59 different ligands in the absence and presence of the ligand lipopolysaccharide (LPS). We discuss how genome-scale signaling models may leverage new types of high throughput data and facilitate personalized medicine.

## Results

### Approximating molecular interactions at steady state

**Fig. 1:**
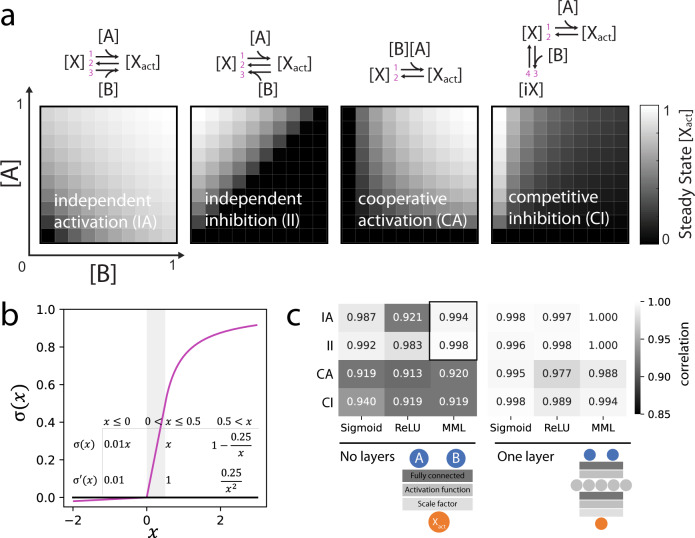
Modeling molecular mechanisms using a feed forward neural network (FFNN). **a** The steady state for different mechanisms involving two source molecules (A and B) at constant concentration and a target molecule with an inactive state ([X]) and active state ([X_act_]). Results were attained by running ODE simulations until steady state, reaction rates (1–4) were parameterized manually (values in Supplementary Table 1), maximum activity was scaled to 1. **b** The Michaelis–Menten-like (MML) activation function is designed as a monotonously increasing, continuous function with a maximum of 1 and continuous first derivative (except at 0). It is composed of three segments; a leaky; a linear; and a saturating. The leaky and linear segments correspond to the leaky ReLU activation function, as the Michaelis Menten equation is not defined for negative input. The saturating segment is composed of a shifted and scaled Michaelis–Menten equation. **c** FFNNs models of molecular mechanisms with different activation functions and number of layers. The model was trained on steady state activity for a grid of 7 × 7 linearly spaced source concentrations and tested on a 20 × 20 grid, the Pearson correlation between prediction and test data was calculated (mean of three runs). A black outline marks the performance of MML for independent activation and inhibition with no hidden layers. Source data are provided as a Source Data file.

For the purpose of the signaling framework developed herein, molecular interactions are assumed to always be at steady state. This can be justified by timescale separation, as these events are expected to occur on the order of milliseconds compared to signal transduction that evolves over several minutes. Molecular dynamics here signifies interactions between signaling molecules through a range of different mechanisms, e.g., phosphorylation, binding, or conformational changes. The steady state assumption implies that the activity of the target molecule of the interaction is a single valued function of its source molecules that are considered constant at that instant. This activity depends on the specific molecular mechanism (Fig. 1a) with the simplest arguably being independent activation and inhibition that may be interpreted as kinases and phosphatases respectively.

In many cases the exact molecular mechanism of a signaling interaction will be unknown, but its input–output relation can be approximated by a neural network. Directed acyclic graphs, i.e., a FFNN, are appropriate models for interactions that are assumed to instantaneously reach steady state^17^ and for independent activation and inhibition, there is a direct mapping between their analytical steady state solution and a FFNN with the Michaelis–Menten equation as activation function (Supplementary Fig. 1a). Based on this we developed a problem-specific activation function, the Michalis–Menten like (MML) activation function (Fig. 1b) with two main features; preventing negative states that would be non-physiological; and preventing states >1 that are non-physiological assuming that this represents full saturation. Physiological constraints are thus imposed at the level of the activation function, allowing weights and biases to take on arbitrary values. In practice, the MML was taken as the leaky version of the Rectified Linear Unit (ReLU) activation in its standard formulation^24^ for negative inputs. This prevents a strict 0 gradient that may cause irrecoverable inactivation of nodes during training leading to blocked signaling in sparse networks. The MML was taken as ReLU also for input values less than 0.5 to allow a range where signaling states can be passed forward without alteration.

We found that a FFNN with this activation function and no hidden layers provided a good approximation of independent activation/inhibition (Fig. 1c) outperforming the other activation functions that were tested, as well as ODEs with incorrect functional form (Supplementary Fig. 1b). The overall performance was acceptable also for other molecular mechanisms, although prediction errors were not uniformly distributed (Supplementary Fig. 1c). An advantage of the MML model without hidden layers was that the sign of weights directly corresponded to the mode of action (MOA), activation (positive), or inhibition (negative). This allows for a straight forward implementation of MOA-constraints. Additionally, it requires markedly less calculations than multilayered FFNNs. For FFNNs with one hidden layer, i.e., intermediate values with no direct biological translation, all of the tested activation functions produced excellent approximations (Fig. 1c).

### Constraining a recurrent neural network with prior knowledge

**Fig. 2:**
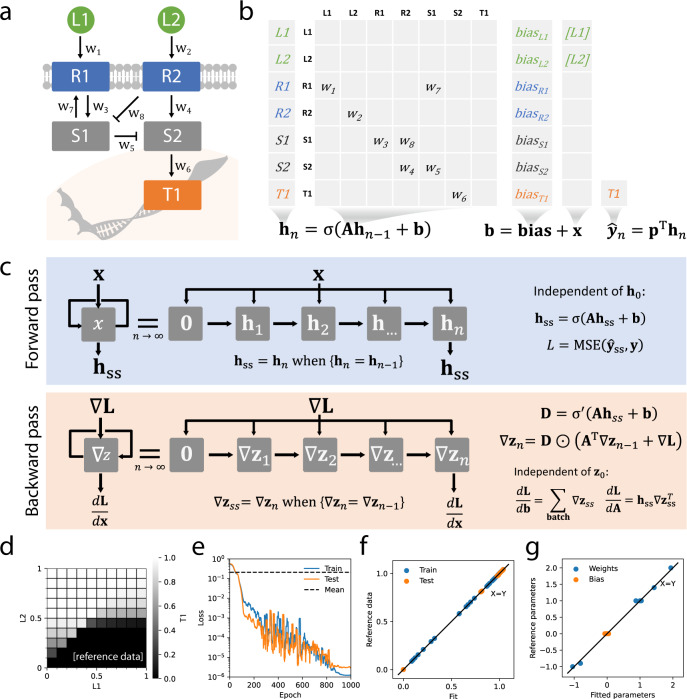
Modeling a simple signaling network using an RNN. **a** A simple signaling network including ligands (L1, L2) receptors (R1, R2), signaling proteins (S1, S2), and a TF (T1) interconnected by 8 interactions (*w*_*1*_*, …, w*_*8*_). **b** The difference equation calculates the state (**h**_*n*_) at timestep n from the previous time step. The matrix **A** holds the interaction weights and the bias term (**bias**) and input term (**x**) are added, the activation function (σ) is applied at each timestep. **c** Calculations are repeated until a steady state (**h**_*ss*_) is reached and the predicted TF activity ($\hat{{{{{{\bf{y}}}}}}}$) is projected (**p**) from the steady state. Loss (*L*) is calculated by comparison to reference data (**y**), e.g., as mean square error (**MSE**). It is back propagated to provide the partial derivatives with respect to weights and biases at steady state. Here ⊙ is element wise multiplication. To prevent potential exploding gradients, the loss is clipped at each step (see methods). **d** Reference data generated by a parameterized model. **e** A model trained on the reference data by minimizing the loss using stochastic gradient descent. Loss from predicting the mean value for comparison (dashed line). **f** Perfect fit (Train) and generalization (Test) to reference data. **g** Parameters in agreement between fit and reference. Source data are provided as a Source Data file.

Signaling involves a network of molecular interactions whose effects propagates from receptors at the surface to TFs in the nucleus. In order to represent these interactions, which include feedback loops, we developed a sparse RNN formulation as a model of cellular signaling, LEMBAS. We constructed a minimal signaling network (Fig. 2a) to demonstrate its application. The structure of this prior knowledge network was encoded by a sparse matrix holding the weights of its molecular interactions (Fig. 2b). The overall expression, also known as a first order non-linear difference equation, iteratively calculates the signaling state from the state at the previous timestep and includes ligand concentrations as input and a bias term, which may be interpreted as basal activation or thresholding. In this study molecular interactions were modeled without hidden layers so that MOA could easily be constrained, but the approximations of molecular interactions could have been made arbitrarily complex by adding intermediary nodes between sources and targets.

It is here assumed that signaling activity reaches a steady state after evolving for some predefined number of timesteps (Fig. 2c) and TF-activities are projected from the steady state. Ligand-concentrations, weights, and biases are all assumed to stay constant during the iterations, which can be motivated by time-scale separation. Often RNNs are used to fit time series or other sequence data, but here intermediate states are discarded resulting in a one-to-one relation between ligand patterns and steady state TF activities. It can be noted that while internally, a trajectory is computed from some initial state (here all zeros) to steady state, the steady state does not explicitly depend on the initial state or any of the intermediary steps and these are therefore not required to reflect biologically relevant transitions. An implication of the steady state assumption is that any oscillations exhibited by the network are dampened.

When training a model using LEMBAS, any potential prediction error can be back propagated to adjust the model parameters. The unrolling of an RNN into discrete timesteps is commonly referred to as backpropagation through time (BPTT)^25^. Here the BPTT expression is simplified by the steady state assumption and the assumption of constant input (Fig. 2c). Due to these assumptions, the gradients only depend on the steady state values and due to the vanishing of gradients from early timesteps, the back-propagated error can be assumed to reach a steady state that is independent from the trajectory by which it is computed (see Supplementary Fig. 2 for a numerical comparison and Supplementary Notes 1 and 2 for derivation). It can be noted that BPTT, for this restricted RNN, strongly resembles loopy belief propagation that is used for Bayesian inference on cyclic graphs^26^, where error messages are propagated until convergence. As an alternative to direct iterations, we also tried using Newton’s method for the forward pass and an equation solver for the backward pass, which is a linear equation system. However, in these tests, the solvers were an order of magnitude slower than iteration (Supplementary Fig. 3a, b).

Models can be constructed on training data, then tested for generalization on previously unseen data. The data, containing ligand–TF activity pairs, was generated from a reference model (Fig. 2d) with manually assigned parameters. A model was trained using this data, i.e., without direct access to the parameter values. Terms were added to the loss function to constrain weights by their MOA and constrain biases at ligand positions to zero, since their concentration was assumed to be provided as input (see methods for implementation). Additionally, regularization terms controlling the L2 norm of the parameters were added to prevent overfitting, as is common practice. The model was trained using the ADAM optimizer^27^ with a cosine learning rate schedule and warm restarts, as has been proposed by others^28^. Using this setup, it was possible to train (Fig. 2e) a model to a near perfect accuracy, both on data used for training (80%), and on test data (20%) that was left out of the training set at random (Fig. 2f). We tested the methods sensitivity to non-uniformly distributed training data by adversely selecting samples that were left out of training (Supplementary Fig. 4a) and this reduced generalization marginally, e.g., removing the bottom left quadrant reduced the correlation (Pearson) of predictions to 0.85.

For this particular model structure, the trained model accurately recovered the original parameter values (Fig. 2g). However, it was possible to construct a network where this did not occur even though the network generalized perfectly to test data (Supplementary Fig. 4b). In this model, with sequential nodes without branching, the parameters were not identifiable, i.e., several different parametrizations provide an equally good fit to the data. Nevertheless, there was a strong correlation between the predicted state vector of trained- and reference-model, suggesting that the learned model may be able to accurately predict the effects of perturbing states, despite inconsistent parametrization.

### The steady state assumption and spectral radius constraints

The assumption of a pseudo-steady state is motivated by time-scale separation; however, this is expected to be disrupted by regulatory events over the course of a few hours, e.g., through transcriptional changes that remodel the network structure or induce autocrine signaling. An example of this is signaling via NFκB, which drives oscillatory TF activities through transcriptional induction of NFκB-inhibitors, e.g., p100 and IκBα^29^. In principle such regulatory effects could be modeled as transitions between two steady states where data on the regulatory change is used as input for the second state, e.g., increased levels of p100 in the case of NFκB signaling. In support of this principle, we constructed a simple model of NFκB dynamics (with 4 molecular species) that elicited oscillatory behavior driven by time delay in protein translation (Supplementary Fig. 5a). By sampling data from the trajectories of this model at 4 selected time points (Supplementary Fig. 5b), and training a steady state model to fit the data (Supplementary Fig. 5c) we found that the parameters of the underlying model were correctly identified (Supplementary Fig. 5d).

**Fig. 3:**
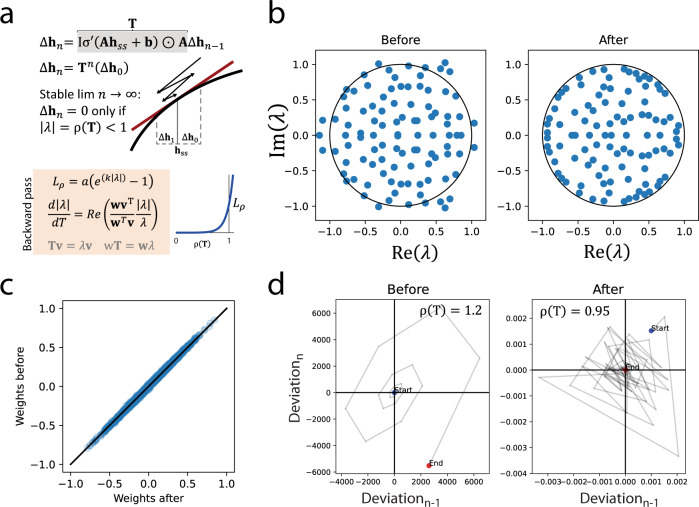
Stability of each steady state is controlled by the spectral radius of a transition matrix. **a** Linearization of the difference equation (first order Taylor expansion) around a steady state (**h**_*ss*_) and shift of coordinates with the steady state as origin yields a homogeneous linear difference equation with transition matrix (**T**), here **I** is the identity matrix. Stability requires that a deviation ($\triangle {{{{{{\bf{h}}}}}}}_{0}$) from steady state tends to 0 for repeated multiplication by **T**. This occurs only if the spectral radius (*ρ*) of **T**, i.e., the eigenvalue (λ) of **T** with largest absolute value, is less than 1. A regularization term (*L*_*ρ*_) is constructed to constrain the spectral radius. Its gradient is a function of the eigen value and left (**w**) and right (**v**) eigenvectors. Note that the imaginary component of this function is orthogonal to the radius and can be ignored. The Re function elementwise returns the real part of complex numbers. **b** All eigenvalues of a 100 × 100 transition matrix before and after a reduction of the spectral radius with the loss function using gradient descent for 200 steps. Matrix parametrized at random with 20% non-zeros. **c** Strong similarity between matrix weights before and after reduction suggesting that the regularization will cause minimal disruption to the learning. **d** Trajectories are stable after shrinking the transition matrix, here the trajectory of the first element. Source data are provided as a Source Data file.

Feedback loops can prevent an RNN from reaching a steady state. The formulation above assumes that a steady state is reached within a specified number of timesteps. However, depending on the parametrization this may not occur. This could yield non-sensical output and may be detrimental to the gradient calculations, which could prevent training from converging. The requirement to reach a steady state can be formally expressed using eigenvalue analysis of the linearized difference equation (Fig. 3a). For the model to eventually reach a steady state, the absolute value of the largest eigenvalue of the transition matrix, i.e., the spectral radius, must be less than 1 (see Supplementary Note 3 for derivation). Similar ideas have previously been explored for linear systems^30^ and for RNNs^31,32^.

It is possible to constrain the spectral radius. Its partial derivatives can be computed (for a numerical demonstration see Supplementary Fig. 6) since it is a locally smooth function of the weights^33^. We introduced a regularization term to control the spectral radius using gradient descent (Fig. 3b) and with marginal effects on the magnitudes of the weights (Fig. 3c) it ensures steady state behavior (Fig. 3d). The introduction of the spectral radius in the loss function can be viewed as imposing a prior on the temporal complexity of the model. It should be noted that while the spectral radius regularization ensures that all conditions in the training data reach a steady state, it does not guarantee that this holds for arbitrary conditions, i.e., untested conditions may be unstable. With this, we had the prerequisites to simulate networks of arbitrary size and wiring.

### Parameterizing a large model for synthetic data generation

**Fig. 4:**
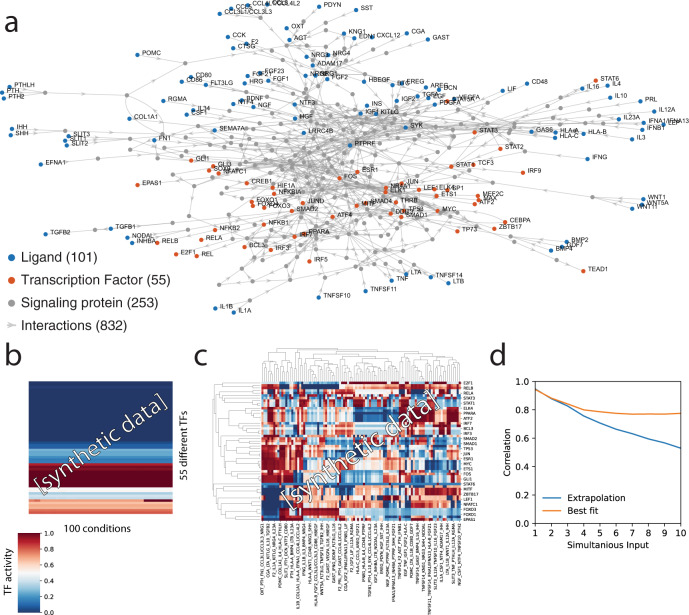
Synthetic data from automatically parameterized model. **a** A large signaling model was reconstructed. **b** Random parameterization did not result in a model where TF activities varied in response to different ligands. Synthetic data (*N* = 100) generated from the model for combinations of 5 different ligands. **c** A model with parameters optimized for variable output resolved this issue. **d** Extrapolation of a linear fit to TF activities from single ligand stimulation and best linear fit using data from multiple ligands (20-fold cross validated) shows that TF activity is not a linear combination of the activity of individual ligands. Due to space constraints, some of the labels are omitted from the heatmap in c. Source data are provided as a Source Data file.

To put LEMBAS to the test, we reconstructed a more comprehensive signaling network. For this, we turned to an online database, OmniPath^9^, that collects evidence of signaling interactions in human cells. The full set of interactions in OmniPath is very comprehensive and includes both well-characterized interactions and results from single high-throughput experiments. To ensure a model of high-quality, we used a subset of interactions that listed KEGG^10^ as reference database and for which the MOA was known (Fig. 4a). Nodes were labeled as ligands, receptors, signaling molecules or TFs, based on annotation in OmniPath (see “Methods”).

We set up a reference model to generate synthetic data. To parameterize such a large model by hand would be daunting, and parametrizing it at random did not yield meaningful models (Fig. 4b), where TF activities depended on the input. This appeared to be a consequence of the biological network structure, since matrices with random structure and similar sparsity showed a more dynamic response when parameterized at random (Supplementary Fig. 7). To overcome this, we devised a setup to automatically generate parameters based on desired properties of the model (see “Methods”), which when applied to the simple signaling network in Fig. 2a, recapitulated its signaling profile without the need for manual calibration (Supplementary Fig. 8). Briefly, using randomly generated input, an objective function was optimized to simultaneously minimize; mean correlation between conditions and TFs; the L2 norm of biases and weights; and deviations from a uniform distribution of activities for TFs and conditions. Additionally, the spectral radius, MOA of weights, and the bias term on ligands were constrained. The resulting model generated much richer synthetic data with TF activity patterns that responded to ligand input (Fig. 4c). Principal component analysis of the models, TF-patterns showed that increasing the number of ligands increased the covered space consistent with complex interactions and emergent states (Supplementary Fig. 9). This was also supported by a generalized linear model of the patterns that showed a decreasing fit for an increasing number of simultaneous inputs, suggesting the presence of non-linear interaction effects (Fig. 4d). The parameterized reference model demonstrates the computational capacity that lays latent in the topology of the signaling network.

The time complexity of LEMBAS is favorable for learning large networks. Network size can be characterized by the number of signaling nodes (n) or by the number of non-zero interactions (z) and a bottleneck for the algorithm involves sparse matrix multiplication between the weight matrix and the state vector with a complexity of zn^2^, meaning that simulation time increases linearly with the number of interactions and that doubling the number of nodes requires 4 times longer simulation time. For biologically relevant networks with between 1000 and 19,000 nodes and ~10 interactions per node, we observe a linear increase in wall time from 0.006 to 0.06 s per pass (Supplementary Fig. 10a). However, the purpose of the algorithm is to train models from data to an acceptable fit, which requires training for a number of epochs that will depend on the total number of conditions (see Supplementary Note 4 for a more in-depth theoretical analysis of the complexity). Empirically we find a sub-linear relationship between the number of conditions and number of epochs of training required to attain a fit (Supplementary Fig. 10b, c). While the complexity of training a generalizable model, so far remains an empirical question, polynomial bounds on the number of epochs have been established for some classification tasks using RNNs^34^.

### Training generalizable models on synthetic data

To test the data requirements for generating generalizable models, we trained models on synthetic data generated from the reference model. To aid in the generalization and prevent the model from getting stuck in local minima during training, several regularization techniques were applied (see “Methods”). Briefly, the state variable was regularized to have approximately uniform distribution and a non-negative max value across conditions; weights were regularized to have non-zero values and L2 regularization was applied to all parameters; Gaussian noise was added to the state variable with the level of noise decayed throughout the training in proportion to the learning rate. Training with noise could be considered a more biologically realistic alternative to drop-out, a regularization technique that aims to decreases the dependency on specific nodes by removing them at random. Experiments with drop-out on knowledge primed neural networks by others^22^ showed that a much lower dropout rate than the default (50%) is required, presumably due to the likelihood of complete blockage when the number of possible paths are limited.

**Fig. 5:**
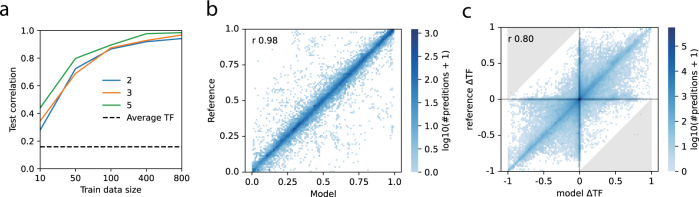
Modest amounts of synthetic data is sufficient to train generalizable models. **a** Synthetic training data was generated from a reference model and used to train an independent model. Training data with different numbers of conditions (10, 50, 100, 400, and 800) and simultaneous ligands (2, 3, and 5). Generalization performance was evaluated on 1000 independently generated test conditions as the Pearson correlation between prediction and reference. A non-zero correlation was attained for predicting the average of each TF. Training was conducted for 10,000 epochs with a batch-size of 5. **b** Comparison of model prediction and reference for the best performing model. **c** KOs of signaling nodes were simulated by applying a strong negative bias to the node, resulting in their state being close to zero after applying the activation function. For independent KOs of 253 internal nodes under 100 random conditions, the change in TF activity for KO compared to no KO (control) was in good agreement between model and reference. For predictions to be off by more than 1 (marked in gray) both KO and control must be incorrectly predicted. Source data are provided as a Source Data file.

With these techniques we fit models that generalized to a quite favorable extent. The amount of data required for this was investigated by training models with increasing amounts of randomly generated conditions for different numbers of simultaneous ligands (Fig. 5a). More simultaneous ligands improved generalization, and excellent performance was attained at the highest data settings. As expected, training models without spectral radius regularization caused training to diverge, resulting in poor fits (Supplemental Fig. 11a). A low, but non-zero, correlation was attained for models trained on data with scrambled order of conditions (Supplementary Fig. 11b). This could be due to the model learning general differences between distributions of individual TFs and was corroborated by an even higher correlation from taking the average of each TFs as prediction. We were concerned about potential information leakage from the reference model, since some of the regularization terms were shared with the parameterization algorithm, but training a model using only regularization terms (without fitting to data) did not perform better than predicting the average of each TF (Supplementary Fig. 11b), suggesting that leakage was not substantial.

For the best model, the predicted TF values generally fell on the line of identity when comparing to reference (Fig. 5b). There were however some notable exceptions, these corresponded to a few poorly predicted conditions with correlations as low as 0.2 whereas the correlations of individual TFs were all above 0.9 (Supplementary Fig. S11c). Training with additional data could potentially alleviate this issue, since a larger state space would be sampled, but the saturating trend in generalization after 400 samples (Fig. 5a) suggests that perhaps further improvements to the regularization may be more economical. We found that, in general, parameters were not identical between reference and trained models (Supplementary Fig. 11d), presumably due to lack of identifiability, but that most of the state variables were still highly correlated between trained and reference models (Supplementary Fig. 11e).

We hypothesized that the fitted model would predict in silico knock outs (KO) of signaling molecules in the reference model without training on such data. If successful, this would mean that the trained models had acquired the same structural dependencies as the reference model. For models trained on data from living cells, this would correspond to the ability to predict systemic effects of mutations or drugs. We simulated KO of each of the signaling molecules under several different conditions, i.e., in presence of different ligands. Although many KOs had limited impact on most TFs, the predicted difference in TF activity was similar between reference and fitted models (Fig. 5c), meaning that KO events were in general successfully predicted.

LEMBAS relies strictly on prior knowledge of the signaling network and does not attempt to identify novel interactions. This is advantageous since it strongly reduces the solution space, alleviating the data requirements while at the same time enforcing biological plausibility and enabling the simulation of KO events. However, this may also be a limitation for learning the parts of signaling that are not yet fully characterized. This may be particularly challenging when applying LEMBAS to other mammals, since their prior knowledge networks often are mere homolog-based extrapolations^9^ of their human counterpart. At best, training under such conditions may result in inability to completely fit the test data, which may help highlight signaling interactions that require further attention and research, however, incorrect relations may also be learned which may harm generalization. A potential solution is to allow the model to use a limited number of interactions supported by prior knowledge of sub-standard quality. Alternatively, the model could be set up to identify novel interactions, which may result in data-driven discovery.

A recent approach attempts to overcome missing signaling interactions by adding condition-dependent signals to each node^35,36^. In theory, missing interactions may be inferred from analysis of the imputed signaling patterns. We adapted a version of this method using a fully connected neural network. We applied it, *post hoc*, to a model from which we had manually removed one interaction (between RALGDS and RALA) that we found was important for the model’s predictions. After training the neural network to reduce the error introduced from removing the interaction, it provided an unambiguous signal on RALA, the target of the interaction (Supplementary Fig. 12a). Furthermore, by identifying nodes that correlated with the signal, and discarding downstream signaling proteins, we narrowed down the source of the missing interaction to a set of 5 nodes that included RALGDS and its nearest neighbors in the signaling network (Supplementary Fig. 12b), supporting the utility of the method. However, for a more realistic setting, where fitting of the model and condition-specific signals occurred simultaneously, we were unable to identify the missing interaction (Supplementary Fig. 12c). So, while data-driven discovery seems like a promising future application of LEMBAS, a more thorough evaluation would be required to determine its utility in a realistic setting.

### Estimating transcription factor activities

Transcription factor activities are increasingly recognized as a natural bridge between signaling and regulation^37^ and in order to apply LEMBAS to actual experimental cell biology data, TF-activities must be estimated for each experimental condition. Since the lifetime of mRNA is expected to be much shorter than regulatory changes in transcription rates^38^, mRNA concentrations can be expected to be proportional to their formation rates and thus reflect the activity of the TFs that regulate their expression. Estimation of transcription factor activities from gene expression of their targets is an appealing method, due to the comparable ease of data generation, that is becoming increasingly utilized^11^. In this study, we used a gene set enrichment-based method, Dorothea^11^, that estimates probabilities of TF activities from mRNA concentrations of their target genes. In the Dorothea-study, the authors quantify their ability to predict changes in TF activities under defined conditions, such as TF knock-out or overexpression, and found that literature-derived TF-target interactions outperformed interactions inferred from high throughput studies. They provide confidence scores between A and E for each interaction in their database, and for this study, we only use interactions with high confidence (A and B). For modeling purposes, one challenge is to relate the statically inferred probabilities of TF activation to different levels of TF activity, e.g., due to time-occupancy at TF binding sites or levels of polymerase recruitment. Nevertheless, it can be expected that some, presumably non-linear, relation exists, which may be approximated by the RNN model.

We applied the method to a transcriptomics dataset from literature^4^ where macrophages were stimulated with 12 ligands in 23 different combinations. There was in general good agreement for the predicted TF-activities among biological replicates from the same condition (Supplementary Fig. 13a, b) and the inferred activity patterns (Supplementary Fig. 14) appeared to largely agree with known biology, e.g., that RELA and RELB are induced by inflammatory ligands such as interferons and lipopolysaccharide (LPS)^39^. Applying LEMBAS to this data, we fit a model with high accuracy, Pearson correlation *r* = 0.96 (Supplementary Fig. 15a). For this, we expanded the model with interactions from an immune-specific resource, InnateDB^40^, and manually added receptor interactions for the ligands used in the study (Supplementary Table 3). Leave-one-out cross validation (LOOCV) of the model’s ability to generalize to unseen data, showed a correlation of *r* = 0.59 (Supplementary Fig. 15b), markedly higher than for models trained on data in scrambled order *r* = 0.11 (Supplementary Fig. 15c). This was even better than for synthetic data of comparable size, perhaps due to denser sampling from a restricted region of the ligand stimulation space.

### Predicting signaling in ligand stimulated macrophages

**Fig. 6:**
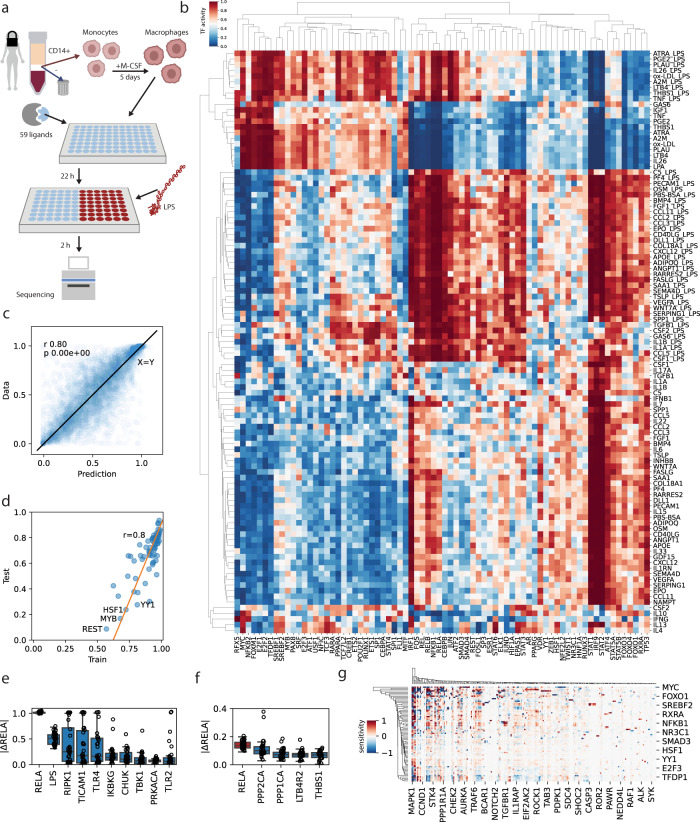
Modeling ligand stimulation in macrophages. **a** Human monocyte derived macrophages were treated with 59 different ligands for 24 h with and without addition of LPS for the last 2 h. **b** TF-activities were estimated for 74 transcription factors across 103 unique conditions (displaying averages across biological replicates). **c** A model consisting of 1262 nodes and 6594 interactions was reconstructed and trained to fit the data. It generalized well to unseen data under cross validation (*r* = Pearson correlation, *p* = two-tailed *p*-value) that included conditions where all ligands were present in at least two conditions (84 out of 103) and included at least once in the train set. **d** The fit of TFs in training data was related to the cross-validation performance. **e** Predicted effect on RELA activity in response to simulated KO under the LPS stimulated condition using the cross-validation folds (*n* = 27) to estimate consistency, top 10 KOs by median effect size, max whisker length 1.5 interquartile range. **f** Top 5 responses to simulated knock in, a red box indicates positive delta. **g** Maximum sensitivity of internal signaling proteins on TF activities across all conditions identified 235 signaling proteins with an absolute sensitivity of at least 0.5 in at least one condition. Abbreviations of none-gene-name-derived ligands, leukotriene B4 (LTB4), all-trans retinoic acid (ATRA), lysophosphatidic acid (LPA), prostaglandin E2 (PGE2), lipopolysaccharide (LPS), oxidized low density lipoprotein (ox-LDL). The boxes in panels **e** and **f** display the median and inter quartile range of the data, whiskers extend to the rest of the data provided that it is within 1.5 inter quartal range of the boundaries of the box. Due to space constraints, some of the labels are omitted from the heatmaps in panel **g**. Source data are provided as a Source Data file.

Encouraged by LEMBAS’s performance on synthetic- and literature data, we generated a data set for ligand stimulated macrophages. Human macrophages differentiated from monocytes from healthy donors (*n* = 3) were stimulated with one of 59 different ligands for 24 h, with and without the addition of LPS for the last 2 h (Fig. 6a). Transcriptomics data was generated for each condition and used to infer TF-activities (Fig. 6b). There was in general good agreement between the biological replicates (Supplementary Fig. 16a, b). As expected, LPS elicited a strong signaling response, inducing, among other TFs, RELA and STAT1. A group of ligands that included IL26 and Prostaglandin E2 (PGE2) showed a signaling profile that was markedly distinct from the PBS control condition and also differed in their LPS response. IL4 and IL13 which are associated with the type II inflammation also showed distinct signaling patterns.

We constructed a model to fit the data. For the signaling network to accommodate the comprehensive set of ligands used in the experimental study it was expanded with interactions from the SIGNOR database^41^, complemented with manual curation of interactions (see “Methods”) and addition of non-protein derived ligands, resulting in a model with a similar number of parameters (1262 biases + 6594 weights = 7856 parameters) and data points (103 samples × 74 TFs = 7622 datapoints). The model fit the data with high accuracy, Pearson correlation of 0.95 (Supplementary Fig. 17a), and generalized well to data that was left out of the training *r* = 0.8 (Fig. 6c) in cross validation with 27 folds (3 conditions per fold). This was markedly better than for models trained on data in scrambled order *r* = 0.19 ± 0.3, *p* = 2.33 × 10^−9^, Mann–Whitney U test (Supplementary Fig. 17a). We also trained models using the leaky ReLU activation function with similar performance *r* = 0.74 ± 0.18 (Supplementary Fig. 17a), but were unable to fit the data using the sigmoid activation function. The generalization performance was poor for 6 of the conditions, suggesting that the model was overfitted in these cases (Supplementary Fig. 17b). The condition with worst generalization performance was SERPING1, which could be related to its complex extracellular activities^42^ that are not covered by the intracellular signaling model. There was a tendency for TF’s that showed a worse fit for the training data to also have worse predictions (Fig. 6d, and Supplementary Fig 17c), suggesting that reconstructing an even more comprehensive network could further improve the predictions.

With the trained signaling models in hand, we applied simulated perturbations to interrogate how predictions were affected by the internal wiring. We focused on the LPS stimulated condition, as this was the ligand we had collected most data for (43 conditions, compared to 2 conditions for all other ligands). We simulated how knocking out each signaling protein would affect RELA (Fig. 6e), a TF that is known to be activated by LPS in macrophages^43^. As could be expected, the LPS receptor TLR4 was predicted to have a large effect, but a number of NF-κB activating proteins were also identified, e.g., RIPK1, CHUK, and IKBKG, in good agreement with prior knowledge^44^. In particular, RIP1 has been found to be a critical modulator of TLR-responsive pathways in human macrophages and has been proposed as a target against chronic inflamation^45^. We also simulated the effect of “knock in”, i.e., activating each signaling protein (Fig. 6f), the predicted responses were generally weaker than for knock out, but the model identified a dampening effect of PPP2CA, which is known to directly dephosphorylate RELA^46^, showcasing that biologically relevant interactions have been learned by the integrative approach. However, the noticeable discrepancies in the predicted effect size between models from different runs, suggests that even larger datasets would be favorable to generate confident predictions of the state of individual signaling proteins.

For a more global analysis of the wiring, we calculated how sensitive each TF was to small perturbations in signaling proteins under each of the studied conditions. From inspecting the maximum absolute sensitivity, i.e., both activating and inhibitory influence, we identified 235 signaling proteins (~20% of the internal nodes) with a strong predicted effect on at least one TF in at least one condition (Fig. 6g). As could be expected, some signaling proteins acted as hubs that affected most TFs, e.g., MAPK1 (also known as ERK2) and GSK3B. Others, only affected one specific TF, e.g., NFIB only affected NFIC. Analogously, some TFs were affected by a large number of signaling proteins, e.g., MYC and FOXO3, while others only by a few, e.g., ELK1 was primarily affected by MAPK1 and JAK2. Applied to a broad range of conditions, sensitivity analysis may be used to identify drug-targets with broad or specific responses. It can also be useful for identifying interactions of high or low importance when constructing smaller scale models of specific processes, localizing non-identifiable parameters^47^, or for network visualization.

### Predicting viability in drug treated cancer cell lines

The main advantage of LEMBAS is that it enables rapid parameterization of large-scale signaling models. For the macrophage data, a model with 1262 signaling proteins was trained and there are few models of this scale available in literature for comparison^48^. One of the most ambitious parameterizations of an ODE-based signaling model in terms of scale consisted of around 1200 molecular species and 2600 reactions^48^. This model was fitted to predict cell viability under the influence of 7 drugs in eight different concentrations in 120 different cancer cell lines (around 6720 observations in total) and used data on basal gene expression and prominent mutations in each cell line to enable context-specific responses. To benchmark LEMBAS against this approach, we adapted our macrophage network, which was of similar scale. To this end we added a projection layer to predict viability as a weighted sum of transcription factor activities, e.g., from MYC and the FOXO and STAT families, mirroring the approach used for the ODE-model (Supplementary Fig 18a). We also added layers that projected signals from drugs to their intracellular drug targets, and mutations and gene expression to their corresponding signaling proteins (Supplementary Fig 18a). Reportedly, the ODE model was fitted in just under a week using a cluster with 400 CPU cores and iterations were halted after 100 steps. The correlation between data and model was then 0.85 ± 0.01 for the fit and 0.69 ± 0.09 for cross validation. In comparison, our framework reached a correlation of 0.87 ± 0.01 within 15 min on a laptop with 6 cores and the cross-validation with a correlation of 0.70 ± 0.06, was completed within an hour (Supplementary Fig 18b, c). Showing that for this task, LEMBAS was comparable in terms of expressiveness and generalization and superior in terms of speed.

## Discussion

We have here demonstrated that genome-scale simulation of intracellular signaling is now attainable. We developed LEMBAS, a computational framework based on RNNs, constrained by prior knowledge of signaling interactions, that rapidly trains predictive models using signal-response data, e.g., TF activities from ligand stimulations or viability in response to drugs. In particular, the model’s ability to predict the effects of KO’s is highly advantageous and cannot easily be matched by black-box-based models. For models trained on real-world data, this would have important clinical implications, since many drugs act by blocking the activity of signaling molecules. We demonstrated the practical applicability of the framework to experimental data, and although our experiments with synthetic data suggest that larger data sets are required to provide even more generalizable and robust predictions, we showed that these models recapitulate known biology and generalize to conditions that were not included in the training. More broadly, this work demonstrates how genome-scale models can enquire consistency between experimental data and prior knowledge and highlight their limitations. We anticipate that LEMBAS in conjunction with large-scale datasets will generate highly generalizable models that further our understanding of intracellular signaling.

Presently, many high-throughput methods are being developed that will synergize well with LEMBAS, including large-scale transcriptomic screenings, e.g., the L1000^49^. These will enable profiling of numerous ligand-combinations and other perturbations to explore the space of possible signaling states and models trained on such data will provide a succinct and actionable representation of the acquired knowledge. LEMBAS is not limited to study ligand-stimulation, our simulations of the effects of drugs on viability and our gene KO-simulations demonstrate how intracellular perturbations can be incorporated. Innovative use of nucleotide barcoding has enabled simultaneous construction of KO cells and sequencing of their gene expression^50^, albeit so far this was only applied to TFs, not signaling proteins. Such intracellular perturbations are of great interest for studying signaling^16,48^ and can help resolve identifiability issues, where activity in multiple pathways can explain the data equally well.

Identifiability issues could be resolved by collecting data on the internal signaling states of select proteins, since in general, observations from more molecular species will improve confidence in the predicted cell states. The coverage of observable vs hidden nodes can be used as a crude metric of this confidence, and this will generally be more challenging for large-scale models that have more molecular species to cover. For example, a smaller scale model of macrophages from literature^51^ covers 70 molecular species with observations for approximately 80% of the nodes in at least one condition, another model^52^ has 130 species with a coverage of approximately 20%. Meanwhile, our model of macrophage signaling includes 1262 species with a coverage of around 5%, although since only around 20% of these had a strong influence on the predictions, the effective coverage after regularization may be higher than this estimate suggests. It should be noted that LEMBAS is not limited to predict TFs, if data is available, any molecular state can be projected from the state vector and fitted analogously to the TF data. High-throughput methods for generating multimodal data are currently under development, e.g., coupled profiling of transcriptome and protein activity^53^ and barcoding states of phosphoproteins using antibodies^54^. Data may also be acquired using proteome level quantification of phosphorylation states^7^.

Transcriptomics is, nevertheless, a strong technology for generating genome-scale data to train signaling models, both in terms of cost and availability. Transcriptome-based TF-activity estimates, provides a much larger set of observables than high level phenotype data, e.g., cell viability^16,19^, which have also been used for fitting large-scale signaling models. The connectivity of TFs throughout the signaling network also helps offset the increase in a number of parameters with increasing network size by a matching increase in number of TFs, i.e., observed datapoints. The use of transcriptomics data to infer TF activity requires reliable estimation-methods. While many activities inferred using statistical methods are of high quality^11^, our understanding of gene regulation is continuously improving and more advanced computational methods are being developed, e.g., auto encoders that fit TF activities as latent variables informed by prior knowledge of TF-gene relations^55^ and mechanism-based deep learning models^38^. There is also the development of sequencing-based methods that simultaneously profile chromatin accessibility, intra-nuclear proteins, and gene expression^56^, which could aid in acquiring more accurate TF activity estimates. There are presently several methods that strive to infer signaling patterns from transcriptomics data and prior knowledge of the signaling- and regulatory networks, e.g., CARNIVAL^57^ and NicheNet^58^. However, these aim to provide qualitative descriptions of possible network wirings for individual condition as opposed to generating a predictive model consistent with all conditions as the one developed in this study.

We here primarily aspired to model the effect of ligand stimulation in a single cell type and differentiation state. This is encoded through the weights and biases. A natural generalization would be to let these parameters depend on external factors, e.g., cell type or test subject. Assuming that the wiring is mostly conserved, these parameters could be made into regularized functions of easily quantifiable properties, e.g., genotypes, allowing personalized parametrizations that still leverage data from other experiments. This was, partially, implemented for the viability case study, where the transcription profile of each cell line affected the bias. Subsets of parameters could even be pretrained using data from molecular studies, which corresponds to transfer learning that has been successful in other ANN applications, e.g., mammograms have been analyzed by appending a classifier to a network pretrained on regular images^59^. This would be particularly useful for analyzing subpopulation-specific responses among cells within a single experimental condition, that are now being inquired with single cell sequencing techniques. It is of much interest to discern the root cause of these differences e.g., differences in ligand concentrations, basal activity, or network wiring. Single cell sequencing has founded an atlas of cell types at various stages of differentiation, and a fruitful continuation of this work, in particular for immune cells^60^, could involve comparisons of their dynamic responses to stimulation through differences between parameterized models.

LEMBAS relies on steady state assumptions motivated by time scale separation. From biological perspective, it seems plausible that evolution would favor reproducible responses, i.e., that a given signaling pattern converges to the same state each time, although there are certainly exceptions, where sustained oscillations are instead desirable, e.g., the circadian rhythm or the cardiac cycle. Due to the steady state assumption LEMBAS cannot directly simulate such dynamics, although internally it calculates a trajectory for each condition. Time-series data could in principle be accommodated by the framework by fitting states at particular time steps, however, this would likely be better accommodated by continuous time RNNs. Such RNNs have obvious similarities with ODE models and discussions on bridging the gap between RNNs and ODEs are ongoing^16^, notably a direct correspondence has been established between RNNs with a specific architecture and a common numerical ODE solver^61^.

Our regularization of the spectral radius ensures that all conditions in the training data reach steady state, but it does not guarantee that this holds for arbitrary conditions. The pursuit of methods to enforce global stability for non-linear systems is an ongoing^62^, but it is not clear if global stability should be required for biological systems, since they may be intrinsically unstable for conditions that are never encountered. Interestingly, if evolution is viewed as an optimization algorithm that has learned cellular parameters from conditions that are encountered, then by analogy turbulent states could be expected to occur for untrained conditions, which may be an interpretation of the chain-of-events in some diseases e.g., the detrimental immune responses known as cytokine storms^63^.

The challenge to learn parameters of a model with known structure from data is not limited to biology. In control theory, it has been proven that SGD can learn linear dynamical systems^64^, which corresponds to an RNN with linear activation function. The RNN developed herein is an example of a sparse ANN. It has been recognized^21^ that for fully connected ANNs trained on image data, most parameters can be set to zero without marked loss in performance. After removing these interactions, the sparse models can sometimes be retrained to the same level of performance as the original, since the learned structure remains encoded in the sparse connections. Analogously for signaling, sparsity has been learned through optimization by evolution. The ongoing development of new algorithms and hardware for training ANNs assures that the future will provide further improvements in model sizes, and training and execution times, e.g., sparse matrix multiplication is parallelizable and can be efficiently calculated on graphic processing units^65^.

There are many avenues to expand the framework to further accommodate realistic simulations. One would be to allow molecules in different cellular compartment to have distinct signaling states. This would add a spatial component to the model and could be implemented directly through the prior knowledge network without changes to the framework. The intrinsic modularity of ANNs allow for intuitive integration with other networks, this seems immediately promising for integration with ANNs of regulatory processes, but it is also conceivable that cell-cell interactions could be modeled by chaining together multiple networks. The use of executable models in cancer research has shown how submodules with varying levels of abstraction can be integrated into a computer program that can be formally verified^66^. The rapid execution of trained models in consort with databases of drug-interaction partners^67^ opens up for genome-wide in silico screening of drug responses. This, together with personalized signaling models could provide individualized predictions of drug responses and side effects at the level of individual cell types.

## Methods

### Ordinary differential equations of molecular dynamics

ODEs were formulated for the different reaction schemas (see Fig. 1a) assuming mass action kinetics (see Supplementary Fig. 1 for an example). The rate constants were manually parametrized (see Supplementary Table 1 for values) to yield sensible output. The differential equations were solved numerically using an initial value problem solver for systems of ODEs (scipy.integrate.solve_ivp^68^ in python 3.7.10). State variables were initialized as 1/[total number of states] and the activity after 100 time units was taken as the steady state value. For convenience, the system was solved once with high resolution, a 50 × 50 linearly spaced grid, and linear gridded interpolation (scipy.interpolate.interpn) was used to down-sample to the indicated operational resolution.

### Neural network simulations of molecular interactions

Neural networks where constructed and trained using the pytorch framework^69^. This includes the autograd functionality, i.e., automatic differentiation, that retains the computation graph and uses it to automatically calculates gradients of the loss function. For the sigmoid activation, the default formulation was used (torch.sigmoid), for ReLU the leaky version was used (torch.nn.functional.leaky_relu), and the MML function was manually implemented (as specified in Fig. 1b). For the fully connected layer (torch.nn.Linear) 5 hidden nodes were used. A trainable scaling factor was added to the output of the functions to accommodate normalization of activities. The neural networks were trained for 5000 epochs using the ADAM optimizer (torch.optim.Adam) with a learning rate of 0.002 and the built in L2 weight decay (factor 10^−5^). Default initialization of weights and biases was used.

### Structure of data files

The signaling network structures were stored in list format with each entry containing a source node, a target node, the mode of action, and references to databases and PubMed ids, where applicable. Signaling nodes were identified by their uniprot identifier. This structure is similar to the format used by OmniPath^9^, but unlike OmniPath, all interactions were considered directed from source to target and reversible interactions were represented by an additional entry with source and target nodes exchanged. The signaling network file was accompanied by an annotation file, that for each of the signaling nodes specified their function, e.g., ligand or transcription factor, and a human readable synonym, e.g., gene name or small molecule acronym. For storage of trained networks pytorch serialized objects (torch.save) were used and a human readable plain text format was also developed where each entry contained the parameter type (bias, weight, input projection, or output projection), parameter value, source node and target node (only used for weights). For the macrophage dataset input and output data for the network were stored as tab separated tables with conditions as rows and ligands and TF levels respectively as columns.

### Projections from input to state and from state to output

Input consists of a [*s* × *i*] matrix where *s* is the number of samples (in total or in the mini-batch) and *i* is the number of ligands in the input, the output consists of a [*s* × *o*] matrix, where *o* is the number of TFs in the output. The RNN calculates a state matrix, [*s* × *n*], where n is the number of state variables. To accommodate size differences between input, output, and state matrixes the RNN is proceeded by a projection layer that inserts the elements of the input at their corresponding position in a zero-padded matrix [*s* × *n*] with elements ordered as in the state matrix. Similarly, the state vector is projected to an output matrix by selecting the corresponding TF elements from the state matrix and placing them in an order that matches the order of TFs in the data. Scaling factors for each element are included in the projections and for the output projection these are made trainable parameters.

### Recurrent neural network formulation

The RNN takes a matrix **x** as input and returns a matrix **h**_*ss*_ as output both with the structure [*s* × *n*], with *s* and *n* defined as above. The function is parameterized by trainable weight and bias vectors. The structure of the signaling network (**A**) is provided as a sparse row matrix (scipy.sparse.csr_matrix) with values of the non-zero elements given by the weight vector. The columns of the matrix correspond to sources and the rows to targets. The state vector is initialized as all 0 and iterated for a finite number of steps, set to 150 in this study. The RNN function was implanted as a manual autograd function (torch.autograd.Function) with both forward and backward pass specified manually (see Supplementary Note 5 for the algorithm) using numpy^70^ operations. The spectral radius of the transition matrix for the backward pass is assumed to be less than 1, meaning that the magnitudes of the back propagated gradients are bounded. However, since it cannot be excluded that this constraint occasionally will be violated during training, gradient clipping is applied at each iteration. To prevent clipping under regular conditions, the clipping function was constructed with a linear segment between two saturating tanh regions (see Supplementary Note 1).

### Initialization of weights, biases, and scaling factors

Weights are initialized as ${{u}}(0,0.1)+0.1$, where *u* is a uniformly sampled random number on the specified interval. Weights corresponding to inhibitory interactions are made negative by multiplication by −1. All weights are scaled by a factor $0.8/{{\rho }}({{{{{\bf{A}}}}}})$, where *ρ*(**A**) is the spectral radius of the matrix **A** to ensure that ${{\rho }}\left({{{{{\bf{A}}}}}}\right) < 1$. Biases are initialized at a value of 0.001 except for biases corresponding to nodes that only have inhibitory inputs, in which case they are initialized at 1 to accommodate dynamic node states in the positive regime. The scaling factors for elements in input and output projections are initialized by a constant value, 3 for input projections (which corresponds to a state of ~0.92 after applying the activation function) and 1.2 for output projection.

### Soft constraints for weight signs and ligand bias

To impose soft constraints, barrier functions were constructed, multiplied by a constant and added to the loss function. For interactions with known mode of action, activation or inhibition, the sign of the corresponding weight was constrained to be positive or negative respectively. This was imposed by adding the sum of absolute values of weights where the sign conflicted with prior knowledge. For biases associated with ligands, the model was prevented from learning large values, since knowledge about ligand concentrations is expected to be available and provided as input. Here, the barrier function was constructed as the sum of squares of biases belonging to ligands.

### State and parameter regularization and application of noise

To aid in generalization and prevent the model from getting stuck in local minima, several regularization techniques were applied. To prevent parameters from taking on extreme values, L2 regularization of weight and bias parameters was implemented by adding the sum of squares of these vectors multiplied by a coefficient, 10^−8^, to the loss function. For training on the synthetic dataset an additional term was added to the weight loss to prevent weights from getting stuck at zero (Eq. 1).

1 $${{{{{\rm{loss}}}}}}=\sum \frac{1}{{w}_{i}^{2}+0.5}$$

Regularization of the state variables was implemented to ensure that they remained active with a wide dynamic range during training, with similar objective as batch normalization. The goal was for each of the elements of the state variable to have a uniform distribution across conditions, and this was implemented by regularizing some of the statistical properties to match the corresponding properties of a uniform distribution on the interval [*a* *b*] (Eq. 2).

2 $${{{{{\rm{\mu }}}}}}\left({{{{{\rm{h}}}}}}\right)=\frac{b-a}{2},\,{{{{{{\rm{\sigma }}}}}}}^{2}\left({{{{{\rm{h}}}}}}\right)=\frac{1}{12}{\left(b-a\right)}^{2},\,{\min }\left({{{{{\rm{h}}}}}}\right)=a,\,{\max }\left({{{{{\rm{h}}}}}}\right)=b$$

To be operational independent of batch size, the properties were calculated across all conditions, and for conditions that were not in the present batch their latest calculated values were used; however, these were detached (torch.tensor.detach) from the computation graph and only gradients from the current batch were back propagated. The regularization was implemented by calculating the deviation of the empirical property across conditions from the ideal property calculated for the interval [0.01, 0.99]. The sum of squares of deviations was applied as barrier function. Additional regularization was added to prevent negative max values, the sum of negative max values was used as barrier function. The sum of these contributions was multiplied by a factor, 10^−5^, and added to the loss function.

In addition to regularization, Gaussian noise was added to the **b** vector for each forward pass, to ensure that the fitted parameters were robust to small deviations. The level of noise was made proportional to the learning rate (lr) as ${{{{{\bf{b}}}}}}={{{{{\bf{b}}}}}}+10\cdot {{{{{\rm{lr}}}}}}\cdot {{{{{\rm{norm}}}}}}\left({{{{\mathrm{0,1}}}}}\right)$, where norm is sampling from a normal distribution with 0 mean and 1 variance.

### Spectral radius regularization

An exponential barrier function was used to constrain the spectral radius (*ρ*) with *k* as scaling factor (Eq. 3).

3 $${{{{{\rm{loss}}}}}}=a\cdot {{\exp }}\left(k\cdot \rho \right),\,a=\frac{1}{{{\exp }}\left(k\cdot [{{{{{\rm{target}}}}}}\,{{{{{\rm{\rho }}}}}}]\right)},\,\left[{{{{{\rm{target}}}}}}\,{{{{{\rm{\rho }}}}}}\right]={{\exp }}\left(\frac{[{{{{{\rm{target\; precision}}}}}}]}{[{{{{{\rm{maximum}}}}}}\,{{{{{{{\rm{number}}}}}}\,{{{{{\rm{of}}}}}}\,{{{{{\rm{steps}}}}}}}}]}\right)$$

To be able to backpropagate this function, we constructed a manual autograd function for the gradient of the spectral radius (see Supplementary Note 6 for the algorithm). It made use of a sparse eigenvalue solver (scipy.sparse.linalg.eigs). Since both left and right eigenvectors are required for the gradient calculation and only right eigen vectors are returned by the sparse solver, the matrix was transposed and solved a second time with the predicted eigenvalue from the first pass as target. To conserve computations, a single steady state was selected at random for regularization from each batch.

### Model reconstruction

The most recent interaction database was retrieved from the OmniPath^9^ website (archive.omnipathdb.org, retrieved 2021-06-21). Only human interactions from the OmniPath core set were included. The interactions were divided into 3 subsets, Ligand-Receptor (LR) interactions, regulatory interactions, and signaling interactions (see supplementary table 3 for details on the queries). The LR and signaling interaction were further reduced to only include interactions that referenced KEGG among the sources. A few reactions were removed based on manual curation; interactions between IL6R and JAK1, STAT3, and SRC were removed, since IL6R only signals through its interaction with gp130 (IL6ST)^71^; the interaction between TLR4 and IRAK4 and CD14 were removed from receptor–ligand interactions, since IRAK4 and CD14 are not considered ligands based on their uniprot annotation^72^. All interactions that were listed as reversible were duplicated and reversed and their interactions were set as unidirectional. To avoid duplicate interactions, all interactions present in LR were removed from the signaling set. Any conflicts in mode of action, i.e., listed as both activating and inhibitory, were resolved by removing the mode of action information. Nodes that were not listed in Uniprot^72^ were also removed. Nodes were classified as ligands if they were listed as sources in LR, as receptors if they were listed as targets in LR, and TFs if they were listed as sources in the regulatory interactions. The LR and signaling interactions were merged. Nodes where considered dead ends and removed from the network if there for the node was no path from any ligand or to any TF. Additionally, nodes were considered redundant and removed if they had only a single source and target that both were the same node. Network plot was drawn using MATLAB 2020a.

The same procedure was followed for the network intended for the literature data, but InnateDB was also included as an approved source. Furthermore, the RL interactions were manually defined (supplementary Table 3) based on the ligands available in the experimental data based on uniprot^72^ annotation. The list of TFs was restricted to the ones with experimental data available.

The same procedure was followed for the macrophage network for experimental data, but SIGNOR was also included as an approved source. RL interactions for non-proteins were manually defined (Supplementary Table 4), and some manual curation was performed (Supplementary Data 1). The list of TFs to include in the network was manually defined.

### Synthetic data generation and analysis

To generate synthetic data with a non-trivial distribution, an objective function with several terms was defined to parameterize the reference model. For each epoch the model was provided with 200 conditions containing five randomly selected ligands per condition with uniformly sampled concentrations, these were resampled for each evaluation. The predicted TF activities were regularized to follow a uniform distribution both across conditions and across different TFs, this was implemented in the same way as state regularization (see above) but without dependency on states from previous epochs. The mean correlations were minimized across both conditions and TFs, this was implemented by calculating the average of the correlation coefficients of the output matrix and its transpose. Spectral radius regularization was applied with a coefficient of 10^−2^ and L2 norm on weights and biases was applied with a coefficient of 10^−6^. Sign and ligand constraints were applied as specified above. To preempt information leakage, parameters were initialized differently than for the trained models; weights were initialized uniformly at random from the interval [0, 3] and their sign was assigned based on mode of action, and scaled to a spectral radius of 0.8; bias was assigned by sampling uniformly from the interval [0, 0.01].

The complexity of the synthetic data was studied by PCA analysis of predicted output from 2000 randomly generated conditions with different numbers of simultaneous ligands. Linear models (sklearn.linear_model.LinearRegression)^73^ were fitted to the synthetic data for each simultaneous ligand level, and prediction performance was evaluated using 20 fold cross validation (sklearn.model_selection.KFold). The performance of the model trained on single ligand data was also evaluated.

### In silico knock outs

The predicted effects of in silico KO were studied by adding a strong negative bias (−5) to the node of interest, resulting in near zero node states after applying the activation function. The change in TF activity compared to the control condition without KO was used as metric since most TFs are not expected to be affected by most KOs. For the KO predictions under the TNF condition, the KO was applied to all nodes in each of the models that were generated for the cross validation, and nodes were ranked by the median of the predicted effect on RELA.

### Sensitivity analysis

For each condition, the reference state without perturbations was simulated. For each signaling protein, the raw state before applying the activation function was calculated. A new simulation was run where each signaling protein was perturbed by an addition of 0.01 times the raw state, this was implemented by adding a matrix with the perturbations on the diagonal to the input. The sensitivity for each TF was calculated by dividing the change in activity by the reference state and multiplying by 100 (1/0.01).

### Inference of TF activity from literature data

Literature data^4^ was retrieved from the ArrayExpress^74^ database (ebi.ac.uk/arrayexpress), accession number E-GEOD-46903. Genes without any detected signal (min *p* > 0.01) or without variance ([std] < 10^−6^ [mean]) were removed from further analysis. The log-transformed data (5203 genes and 384 samples) was centered and TF activities were inferred using Dorothea^11^, as is provided through the R package “dorothea”^75^, which uses an enrichment-based statistical method, viper^76^, with the default settings,. Only TFs with a confidence score of A or B and interacting with at least 5 genes were included. Conditions were filtered to only contain data from GM-CSF cultured macrophages from the same time point (72 h) amounting to 103 samples including biological replicates. The Dorothea reported log odds ratios were transformed to probabilities using the inverse logit function, i.e., the logistic function. The average was taken among replicates resulting in 23 unique conditions. Standard deviation among replicates for each TF within each condition were inspected and TFs were discarded if their 75th percentile of standard deviations exceeded 0.2 corresponding to 4 TFs (see Supplemental Fig. 13a).

### Viability predictions

LEMBAS was appended with additional layers to support viability predictions in different cell-lines using data on basal gene expression, prominent mutations, and concentrations of different drugs. The perturbations of the intracellular signaling state were estimated by incorporating signal projection layers for drug concentration, cell-line gene expression profile, gene mutations, and appending a layer for predicting viability from TF activities (see Supplemental Fig. S18a). The input to this modified model consisted of three types of data: (i) a [*C* × *D*] matrix containing the log-transformed concentration of drugs, where *C* is the number of samples and *D* is the number of different drugs in the experiment, (ii) a [*C* × *S*] matrix containing the basal gene expression of the different cell-lines (log transformed and z-scored) for each signaling molecule in the model, where S is the number of signaling molecules in the network, and, finally, (iii) a [*C* × *M*] matrix containing the genes mutated in each cell-line, where *M* is the total number of unique mutations. Each of the input types were processed by a separate layer and projected to vectors that were additively combined to form a perturbation of the signaling state of LEMBAS.

The drug layer, projects the input (concentrations of drugs) to the targeted signaling protein in the network. Specifically, the input is multiplied by a sparse [*D* × *T*] matrix, where *D* is the number of drugs in the experiment and *T* is the number of known targets. The matrix contains non-zero weights only in positions corresponding to known interactions between a drug and its respective targets^48^. The multiplication product is then projected into the signaling nodes of the prior knowledge network. The gene expression data is projected, using a set of trainable weights and bias terms, and clipped to the interval [-inf 0] by a non-linear activation, in this way adding negative bias to the node states of lowly expressed signaling proteins, the state of highly expressed genes unaffected. The mutations for each cell-line are added to the perturbation signal by through projection via a [*S* × *M*] matrix, where *S* is the total number of signaling nodes in the network and *M* is the total number of targeted/mutated genes, after first being multiplied element-wisely by trainable weights. Finally, the signaling output of LEMBAS ([*C* × *S*]) is projected to transcription factors actives that are linearly combined to predict cell viability through a weighted sum of the transcription factor activities plus bias.

### Cell culturing

Deidentified buffy coats from healthy human donors were obtained from MGH Blood Center. PBMCs were isolated from buffy coats by density-based centrifugation using Ficoll (GE Healthcare). Monocytes were isolated using a CD14 positive selection enrichment kit (STEMCELL Technologies). Isolated monocytes were differentiated to M-CSF-derived macrophages in RPMI 1640 (Thermo Fisher Scientific) supplemented with 10% heat inactivated FBS (Thermo Fisher Scientific), 10 mM HEPES, and 2 mM L-glutamine. Media was further supplemented with 50 ng/mL M-CSF (Biolegend, MCSF: #574802). Monocytes were cultured in low-adhesion tissue culture flasks (Corning) for 5 days.

### Cell stimulation and RNA-sequencing

Cells were detached and re-plated on day 6 into regular attachment 96-well tissue culture plates with 50,000 cells in 100 μL fresh media. Cells were incubated for 24 h for reattachment. To stimulate cells, media supernatant was removed and 98 uL of fresh media was added with 2 μL of designated ligand at 50× concentration. For a subset of conditions, 10 ng/mL of LPS was added to each well 2 h prior to the lysing cells. After 24 h, the supernatants were removed and cells were lysed in 50 μL of RLT lysis buffer (Qiagen) + 1% beta-mercaptoethanol. Lysates were spun down, transferred to PCR plates, and snap-frozen on dry ice prior to library preparation. RNA-seq was performed using a modified, automation-enabled version of the Smart-seq2 protocol with 5000 cells or 5 μL of lysate as input used previously^77^. RNA was isolated using 2.2X SPRI clean-up (Agencourt). Beads were resuspended in dNTP mix, RNase inhibitor (Takara), nuclease-free water, and 3′ RT primer (IDT, 5′-AAGCAGTGGTATCAACGCAGAGTAC(T30)VN-3′) and incubated for 3 min at 72 °C. For reverse transcription (RT), samples in a total of 10 μL were incubated for 42 °C for 90 min, followed by 10 cycles of 50 °C for 2 min and 42 °C for 2 min, and finished with 70 °C for 15 min. RT mix contained Maxima RT (Thermo Scientific), Maxima buffer (Thermo Scientific), Betaine (Thermo Scientific), MgCl2 (Invitrogen), RNase inhibitor (Takara), and TSO (IDT, 5′-AAGCAGTGGTATCAACGCAGAGTACrGrG+G-3′). cDNA was then processed using Kapa HiFi HotStart (Roche) and ISPCR primer (IDT, 5′-AAGCAGTGGTATCAACGCAGAGT-3′) for 15 cycles. cDNA was purified by a 0.8X SPRI clean-up and concentrations were determined using the Qubit hsDNA kit (Invitrogen). Libraries were generated using the Nextera XT kit per the manufacturer’s instructions but with miniaturized reactions. Final libraries were purified using 0.9X DNA-SPRI beads (Agencourt). Agilent bioanalyzer 2100 was used to determine cDNA and library size distributions. All libraries were sequenced using 38 × 38 paired-end reads on a NextSeq 500 (Illumina).

Machine output was converted to FASTQ files using bcl2fastq v2.20.0. The Smart-seq2 Multi-Sample Pipeline (RRID:SCR_018920) was used to generate count and TPM matrices using the hg38 reference. For each cell in the batch, paired-end FASTQ files were first processed with the Smart-seq2 Single Sample v5.1.1 Pipeline (RRID:SCR_021228). Reads were aligned to the GENCODE human (V27) reference genome using HISAT2 v2.1.0 with default parameters in addition to --k 10 options. Gene expression was calculated using RSEM v1.3.0’s rsem-calculate-expression --calc-pme --single-cell-prior. QC metrics, RSEM TPMs and RSEM estimated counts were aggregated into a single Loom file for downstream processing. Count matrices are deposited in Gene Expression Omnibus (ascension number GSE202515).

### Transcriptomics filtering and normalization

The transcriptomics data were quality filtered to only include samples with a total number of estimated reads above 2 million, and a total number of detected genes above 5000. Of the 383 samples, 325 fulfilled this requirement. After filtering, technical replicates were collapsed, resulting in 190 biological distinct samples. Sample specific scaling factors were estimated using DEseq2 (estimateSizeFactors)^78^ and data was variance stabilized (vst) and centered. The effect of donor was regressed out using a function from the limma^78^ package (removeBatchEffect). Dorothea was applied to the resulting data as for literature data as specified above.

### Statistics and reproducibility

A neural network model, **y** = *f*(**x**), was trained on data from ligand stimulated macrophages. Cells were stimulated with 59 different ligands in the presence and absence of LPS, where the absence/presence of ligands for each experimental condition comprised the **x** matrix. Transcriptomics data was collected for each condition and transcription factor activity was derived from the data, comprising the **y** matrix. Samples were excluded if an insufficient number of transcripts (<2 × 10^6^) or genes (<5000) were detected. Model performance was evaluated by the Pearson correlation between prediction and data under cross validation, i.e., data was divided into folds of non-overlapping train and test sets, and performance on the test sets was evaluated for models fitted on the corresponding train sets. The hypothesis that the model could learn generalizable relations between **x** and **y** was tested by comparing the test performances to models fitted to train sets with **y** in scrambled order, i.e., with disrupted relations between **x** and **y**. The statistical significance of the difference between these distributions of correlation values were calculated using the two-sided non-parametric Mann–Whitney U test. No statistical method was used to predetermine sample size, however sample size requirements estimated by simulations on synthetic data (Fig. 5a) indicated that 100 experimental conditions could be expected to yield a predictive performance of around *r* = 0.8 on test data. No attempts were made to reproduce the transcriptomics data. The experiments were not randomized. The Investigators were not blinded to allocation during experiments and outcome assessment.

### Hardware for simulations

Simulations were performed on a Dell Precision 3530 laptop with an Intel i7 CPU @ 2.60 GHz with 6 cores (12 logic processors) and 16 GB ram. For convenience, evaluation of data requirement and cross-validation was carried out on a singled threaded computer cluster (Intel Xeon CPU @ 2.60 GHz) that allowed job scheduling (using Slurm) with 16 parallel jobs.

### Reporting summary

Further information on research design is available in the Nature Research Reporting Summary linked to this article.

## Supplementary information

Supplementary Information

Description of Additional Supplementary Information

Supplementary Data

Reporting Summary

## Data Availability

The count matrices for the 60-ligand RNA-sequencing dataset generated in this study have been deposited in the Gene Expression Omnibus under accession number GSE202515. The OmniPath interaction database used in this study is available through their website (archive.omnipathdb.org/omnipath_webservice_interactions__20210621-20211113.tsv.xz). The transcriptomics data for ligand stimulated macrophages from Xue et al. 2014^4^ used in this study is available at ArrayExpress under accession code E-GEOD-46903 [ebi.ac.uk/arrayexpress/experiments/E-GEOD-46903]. The processed cell viability data and cell line mutation profile from Fröhlich et al 2018^48^ used in this study is available on Zenodo (10.5281/zenodo.1472794). The associated basal cell line expression data (RPKM) used in this study is available at the depmap portal under CCLE 2019 (depmap.org/portal/download). Source data are provided with this paper.

## Source data

Source Data

## Code availability

The code and scripts to reproduce the simulated data are made available through a public GIT repository (github.com/Lauffenburger-Lab/LEMBAS, 10.5281/zenodo.6532706).
