# Supplementary material for: Artificial neural networks enable genome-scale simulations of intracellular signaling
Source: Nat Commun. 2022 Jun 2;13:3069. doi: 10.1038/s41467-022-30684-y (PMC9163072; doi:10.1038/s41467-022-30684-y)
Supplement: Supplementary file 1 — Supplementary Information [file 41467_2022_30684_MOESM1_ESM.pdf]

Supplementary Information

Artificial neural networks enable genome-scale simulations of intracellular signaling

Avlant Nilsson, Joshua M. Peters, Nikolaos Meimetis, Bryan Bryson and Douglas A. Lauffenburger

Supplementary Figures

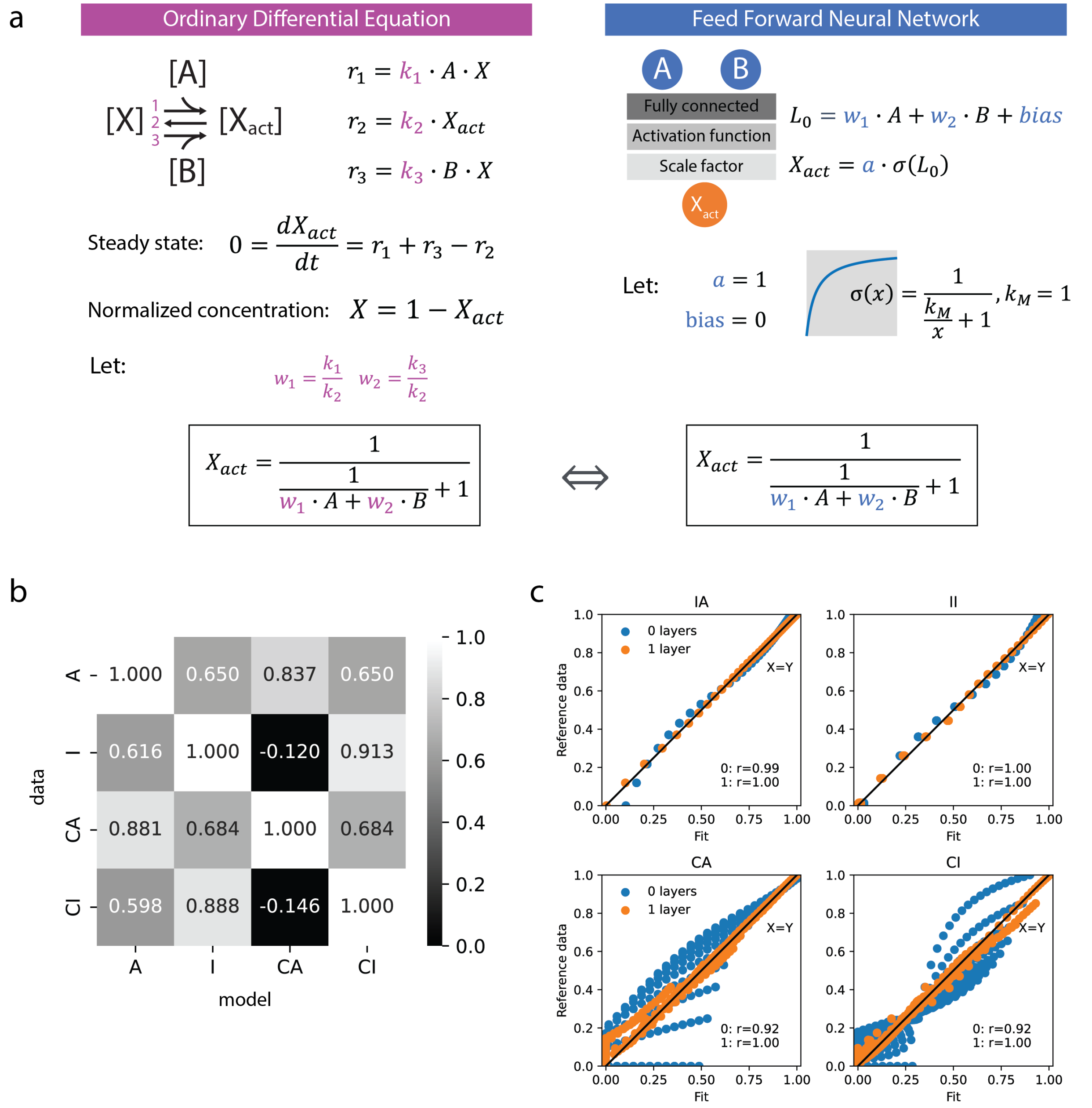

Supplementary Figure 1 Fitting ODEs with a Michaelis Menten like (MML) activation function. a) The analytical solution of steady state activity for independent activation derived from the ordinary differential equations. The equation is equivalent to the equation for a zero-layer neural network with the Michaelis Menten equation as activation function. **b)** best fit of the different ODE formulations to the ODE data, suggesting generally poor fits to data from other functions. ODE parameters were constrained to the interval [0 1]. **c)** Neural network approximations of ODEs with different mechanism using an MML activation function, IA=independent activation, II=independent inactivation, CA=cooperative activation, CI=competitive inhibition.

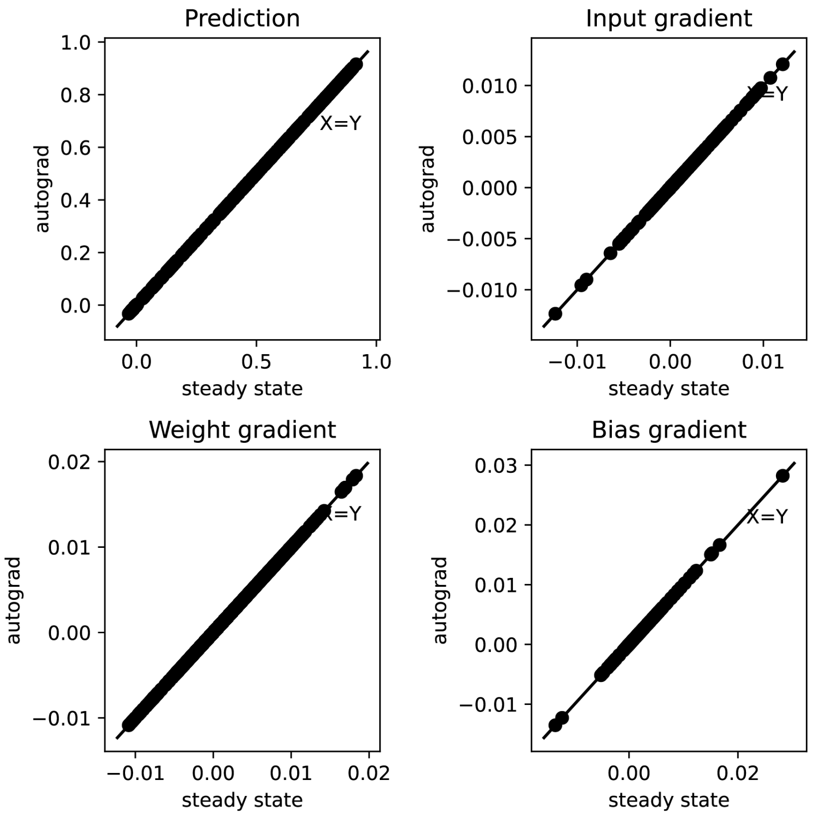

Supplementary Figure 2 Numerical comparison of auto-grad and steady-state for a random network. The predictions and the gradients for input, weights and bias are indistinguishable between models computed by pytorch autograd (automatic differentiation) function and the manually implemented steady-state function. This function only depends on the steady state gradients and thus has significantly lower memory overhead compared to autograd that stores the full computation graph in memory and consequently, autograd takes approximately 10 times longer to complete. This also means that arbitrarily many timesteps can be executed without running in to memory issues.

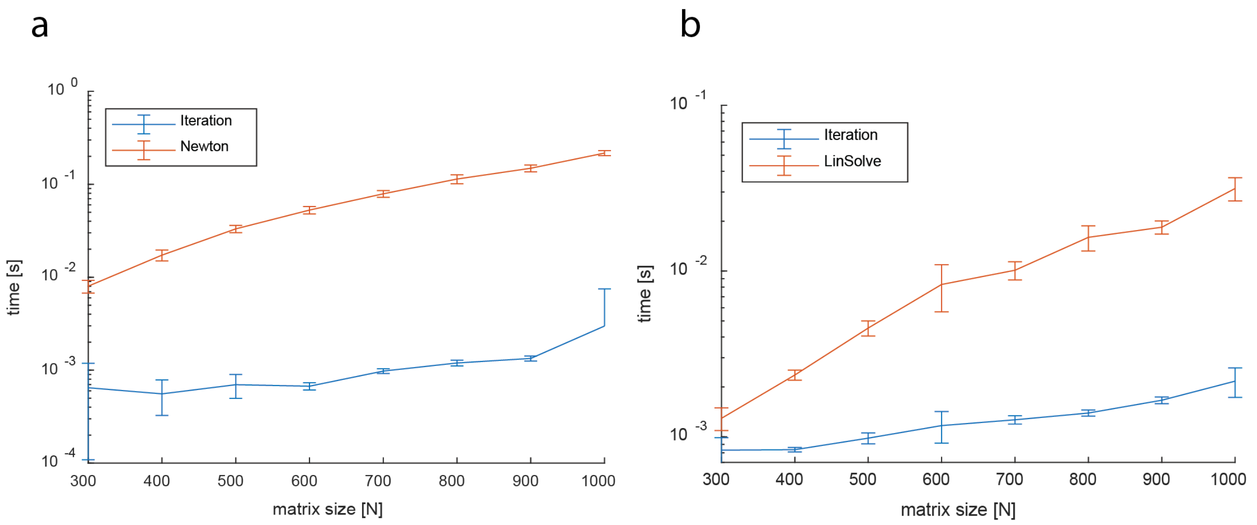

Supplementary Figure 3 Alternative methods for solving the forward and backward pass. a) Newtons method compared to iteration for forward pass, random sparse matrixes of different N with a density of 0.01. **b)** Linear equation system solver compared with iteration for forward pass. Error bars show standard deviation for 10 randomly sampled matrixes at each N.

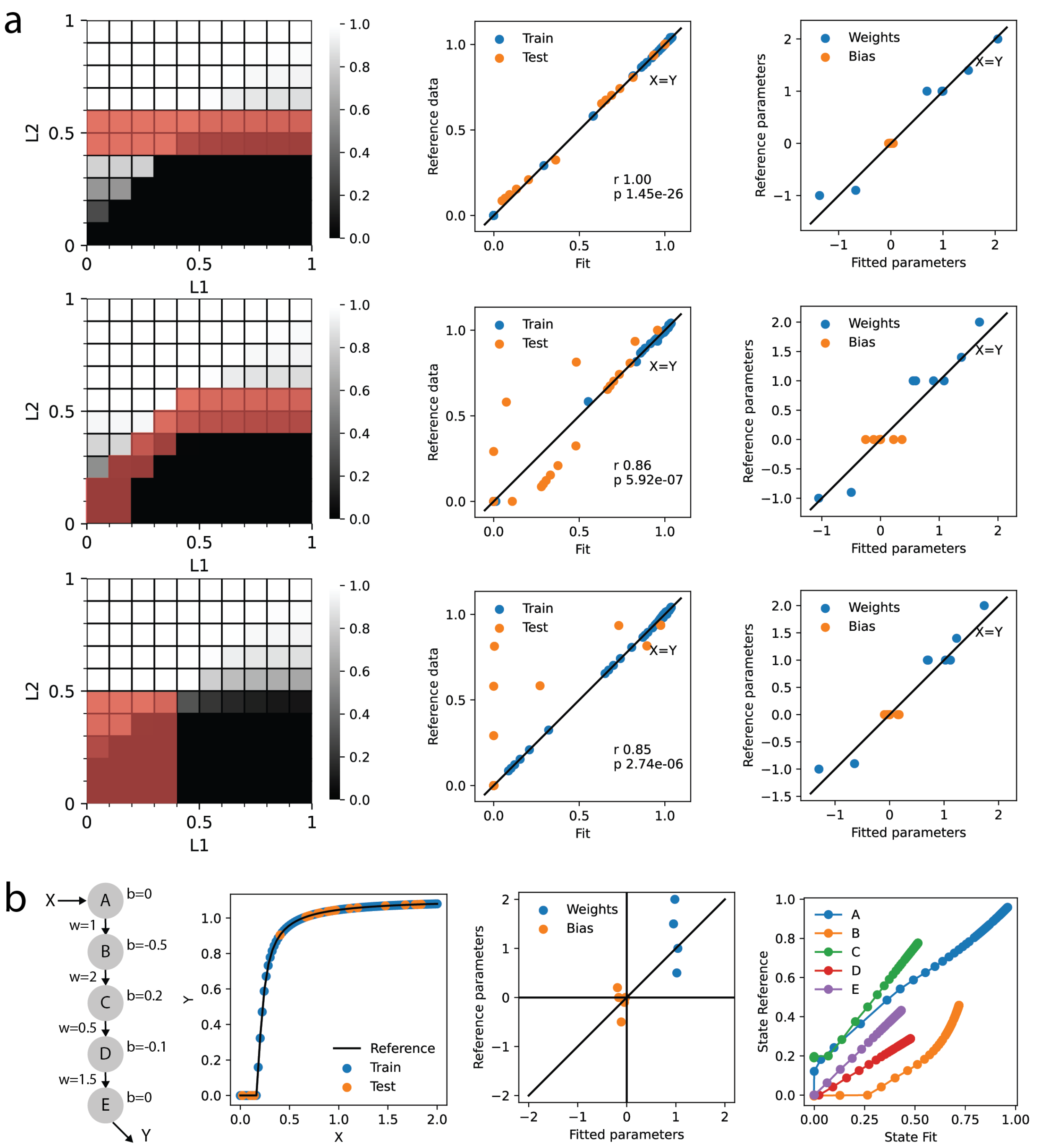

Supplementary Figure 4 Adversarial test data and network structure. a) The networks ability to extrapolate was test by purposefully selecting training data in challenging locations, including along the boundary and in the whole bottom left quadrant. **b)** An unbranched network structure was constructed and a reference model was manually parameterized. The test fit to this model was perfect, but parameters were not accurately predicted. The node states of the fit and reference were correlated. Pearson correlation (r) and the two-tailed p-value calculated using scipy.stats.pearsonr.

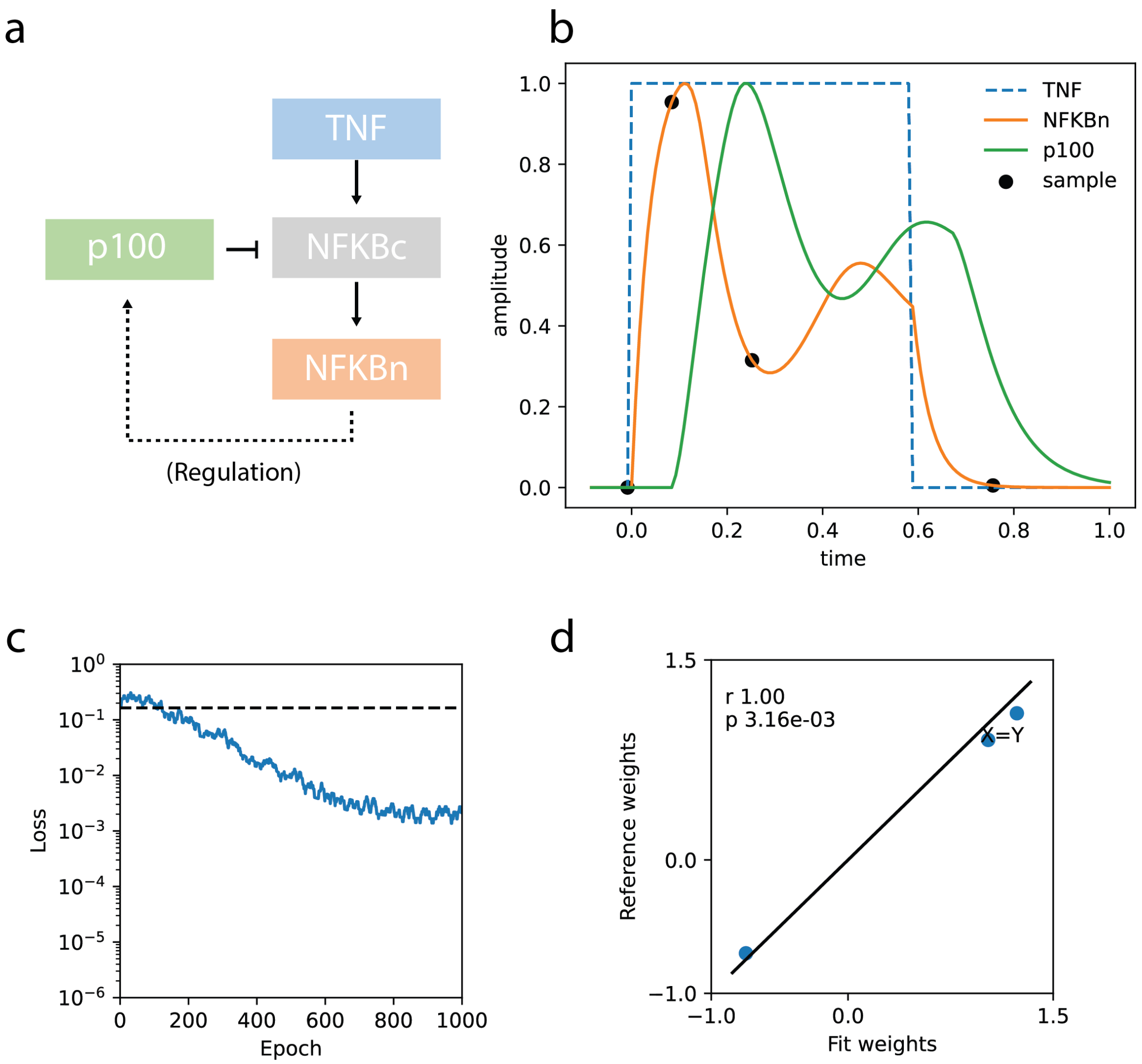

Supplementary Figure 5 Assuming steady state in a dynamical model. a) A minimal model of NFKB activation by TNF with delayed synthesis of p100. **b)** Dynamics of the molecular species and sampling points. **c)** training of model assuming that each sample represents a steady state. **d)** fitted vs actual parameter values. Pearson correlation (r) and the two-tailed p-value calculated using scipy.stats.pearsonr.

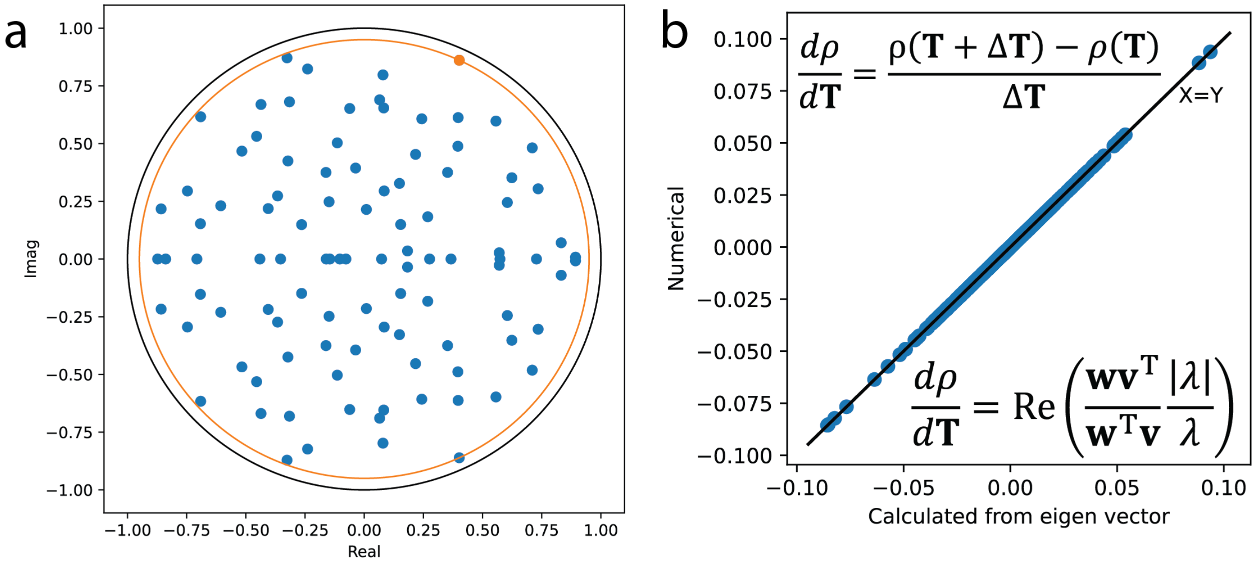

Supplementary Figure 6 Derivative of spectral radius. a) Eigen values for a randomly parameterized transition matrix, where the largest absolute value of the eigen values (orange dot) is the spectral radius. Note that due to the transition matrix being real and non-symmetrical eigenvalues are mirrored along the origin of imaginary axis. **b)** Comparison of the derivative of the spectral radius calculated from its left and right eigen vectors and numerically calculated by perturbing each element of the transition matrix in turn by a small value (epsilon= 10^{-10}) and re-calculating the eigen values.

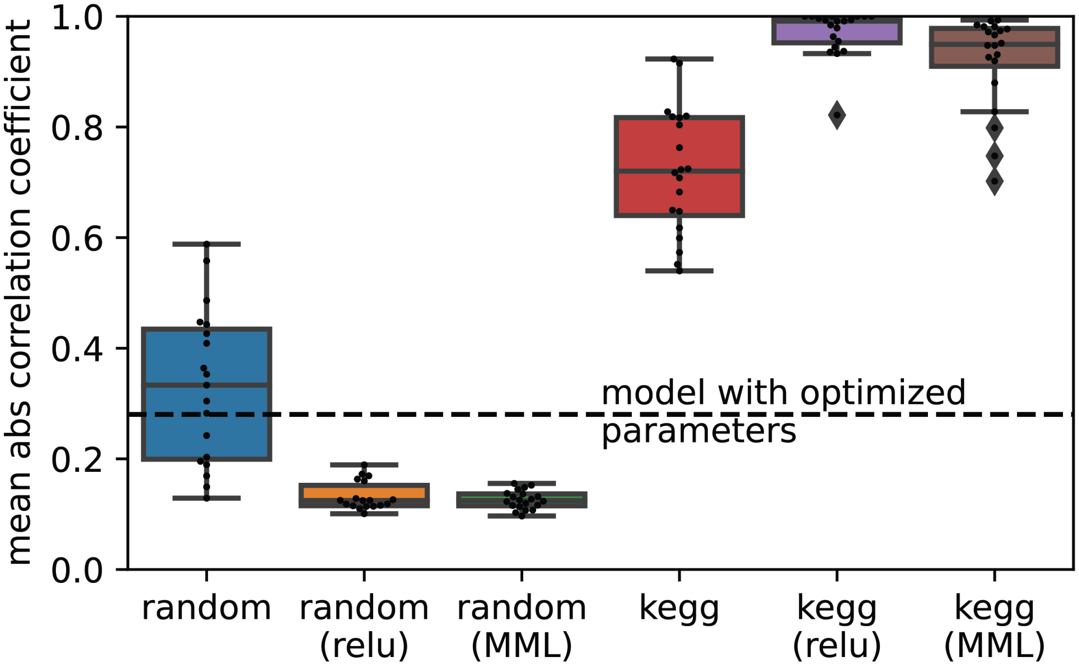

Supplementary Figure 7 Variability in output depending on input for random and biological networks. The variability of the output of a model after 150 recurrent iterations with sparse random networks (N=20) or the KEGG network with random parameterization, without activation functions or with leaky ReLU or MML activation. The variability of the optimized model included for comparison (dotted line). The boxes display the median and inter quartile range of the data, whiskers extend to the rest of the data provided that it is within 1.5 inter quartal range of the boundaries of the box.

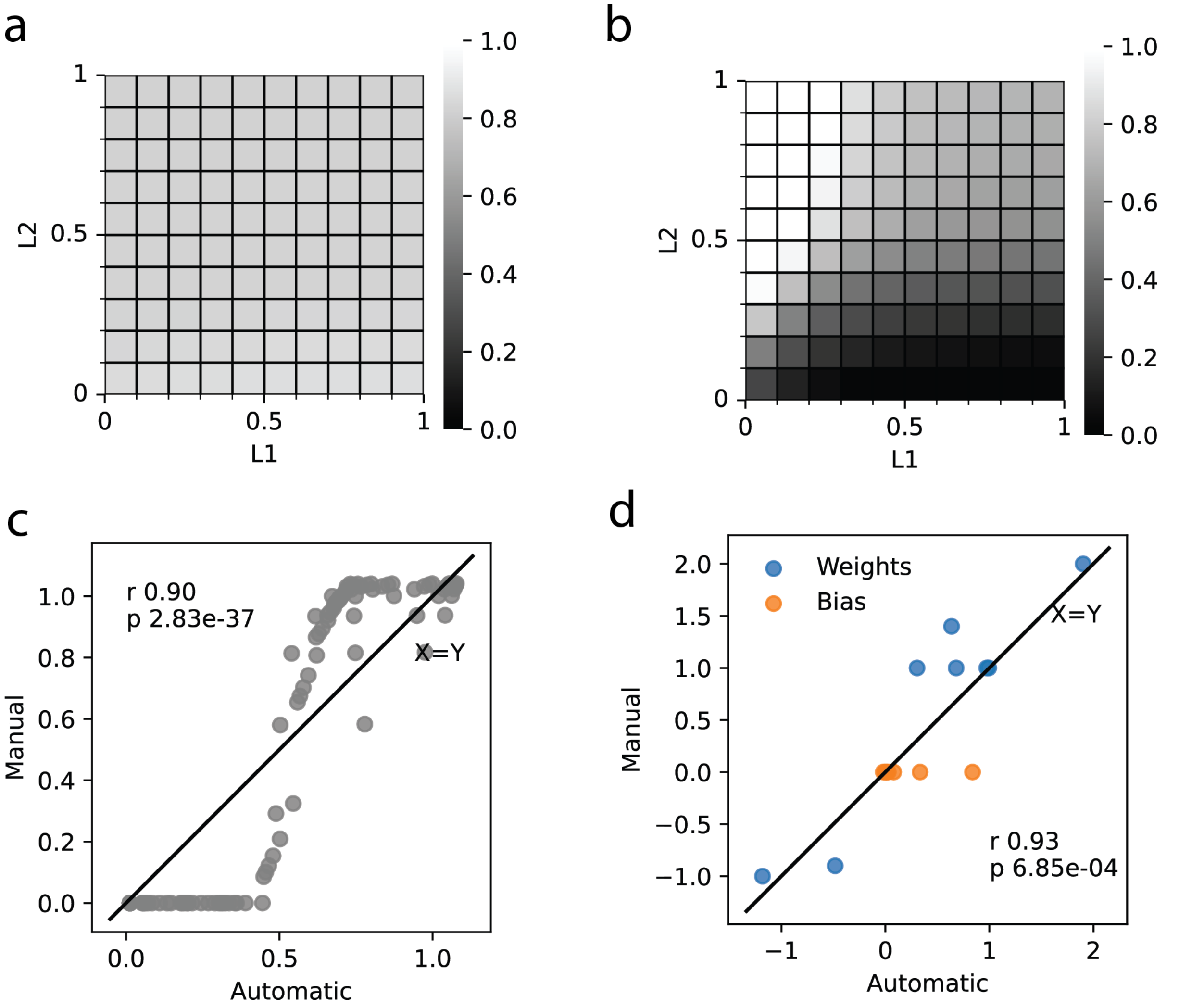

Supplementary Figure 8. Automatic parameterization of the small signaling network. a) output of network parameterized with random weights with same mean (0.48) and standard deviation (0.89) as the manually assigned weights. **b)** output with optimized parameters. **c)** The output between automatically and manually parameterized model is significantly Pearson correlated (r), **d)** The weights for automatic and manual parameterization are significantly Pearson correlated (r). The two-tailed p-value for r calculated using scipy.stats.pearsonr.

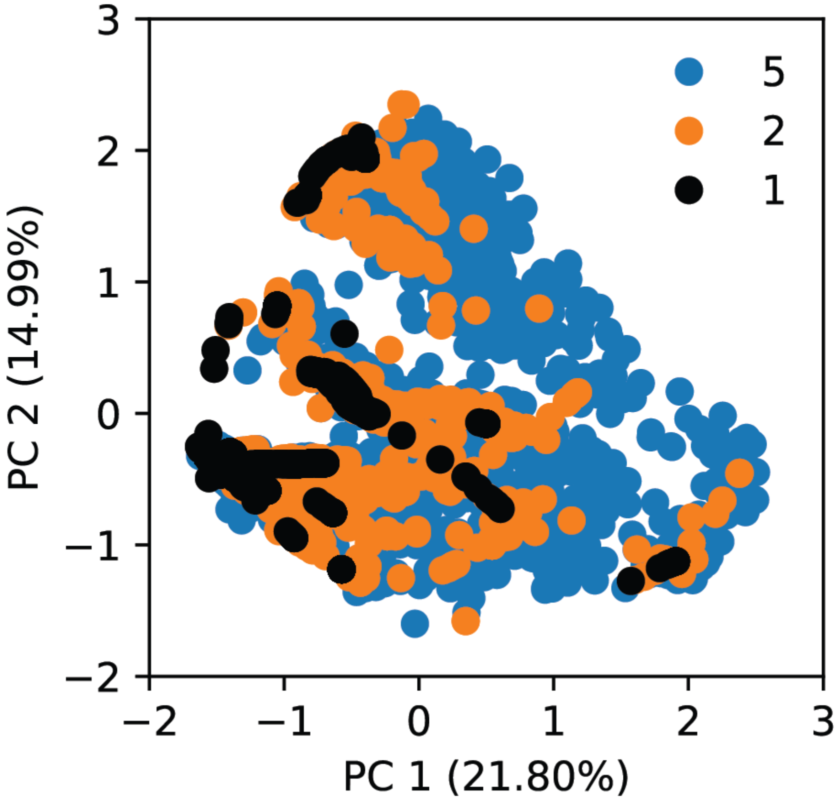

Supplementary Figure 9 Principal component analysis of synthetic model output. Increasing the number of simultaneous ligands (1, 2 and 5) increases the area of TF-patterns in principal component space, 2000 randomly sampled conditions per combination-level.

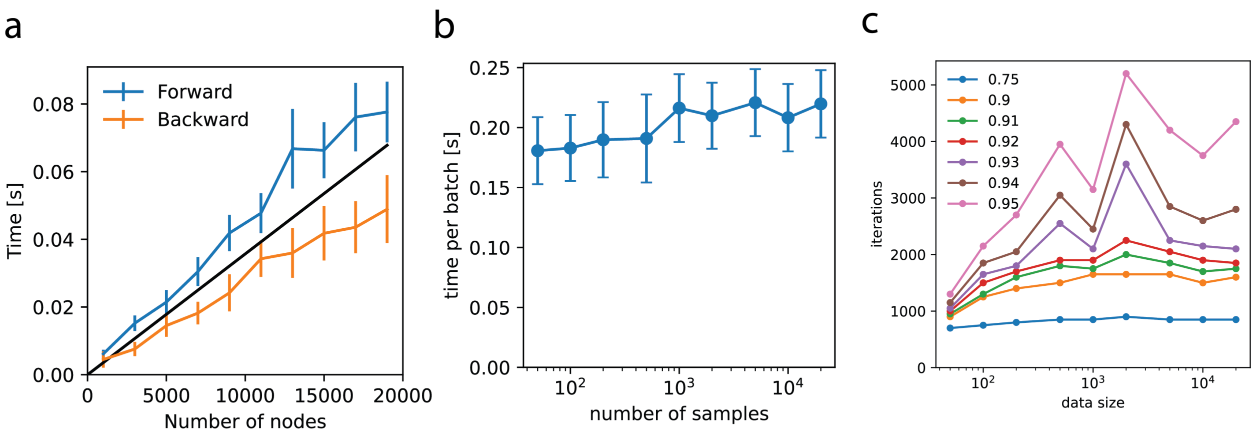

Supplementary Figure 10 Empirical tests of time complexity a) Test of wall time for different network sizes. Sparse random networks with 10 interactions per node were generated (using scipy.sparse.random) and their spectral radius was constrained to 0.9. The average wall time (of 10 random networks) for the forward and backward pass was calculated using random input and output (with batch size of 3) and 100 steps. Error bars show standard deviation. There was a linear fit (R2=0.91) between number of nodes and time. **b)** increasing the total number of samples does not markedly increase the wall time per batch when using a constant mini batch size (n=50), error bars show standard deviation across 10000 batches. **c)** The number of iterations required to reach different cutoffs for convergence for different data sizes increases sub-linearly (logarithmically) with the number of samples for different levels of convergence (correlation between data and fit in legend).

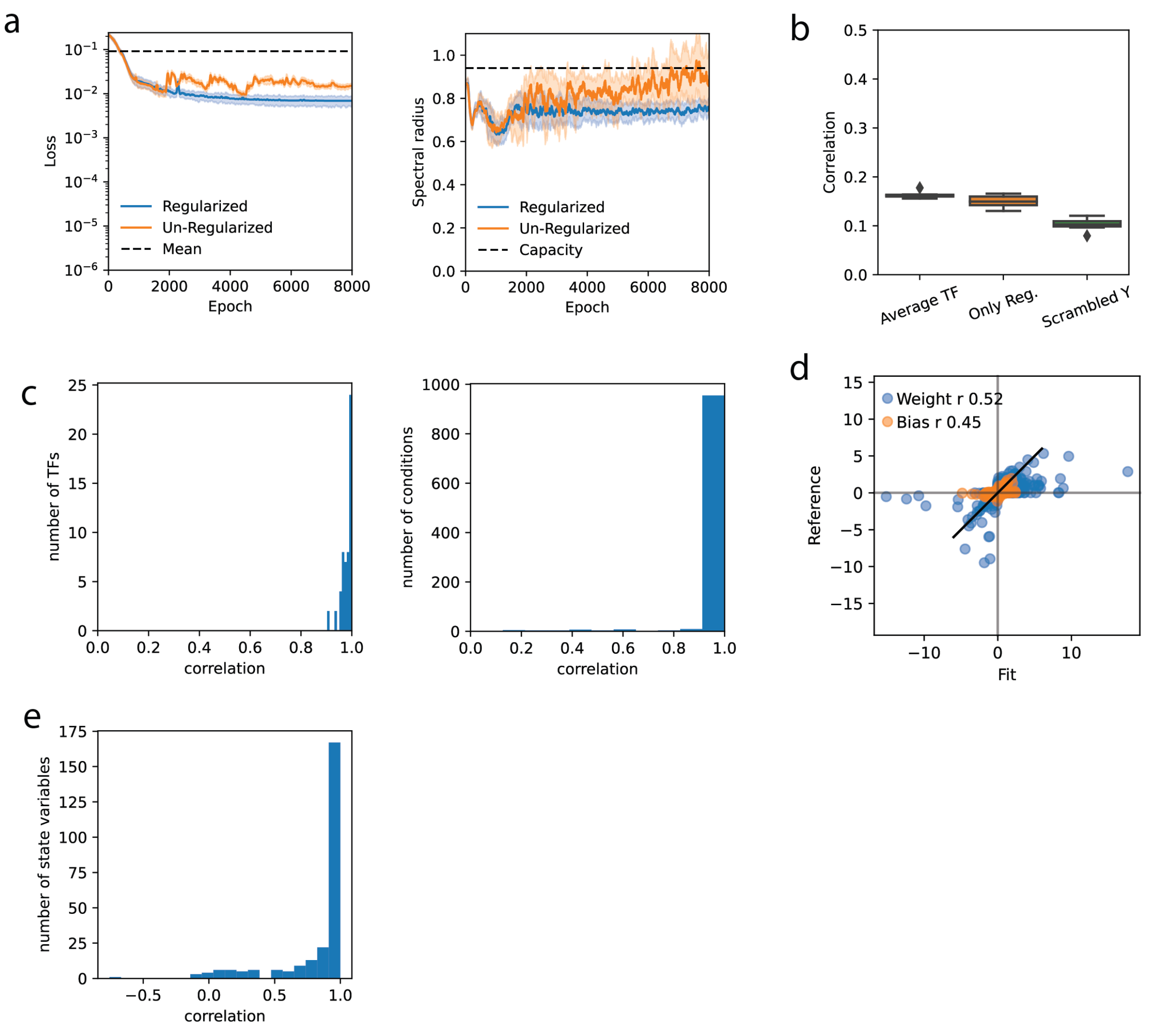

Supplementary Figure 11 Training performance on a synthetic dataset. a) Training trajectories for two models, one with and one without spectral radius regularization trained on data from the same 10 conditions. Without spectral regularization training diverges (around epoch 2000) and the spectral radius sometimes increases above capacity, which is a value (less than 1) that depends on the number of timesteps. **b)** Comparison of fits of a prediction consisting of the average of each TF, a model trained using only the regularization (Reg.) terms, and a model trained with scrambled condition order. 10 tests consisting of 1000 conditions each were sampled at random. **c)** Correlation between model and reference for individual TF and individual conditions evaluated on 1000 test conditions. **d)** Comparison between reference and fitted parameters, note that the correlation for weights is heavily influenced by the sign that is constrained by regularization. The correlation for the absolute value that is not influenced is 0.36, p 10^{-27}. **e)** Mostly high correlation between states of model and reference, here only internal nodes, i.e. excluding ligands and TFs, are evaluated since they are not directly provided by the data. The boxes in panel b display the median and inter quartile range of the data, whiskers extend to the rest of the data provided that it is within 1.5 inter quartal range of the boundaries of the box.

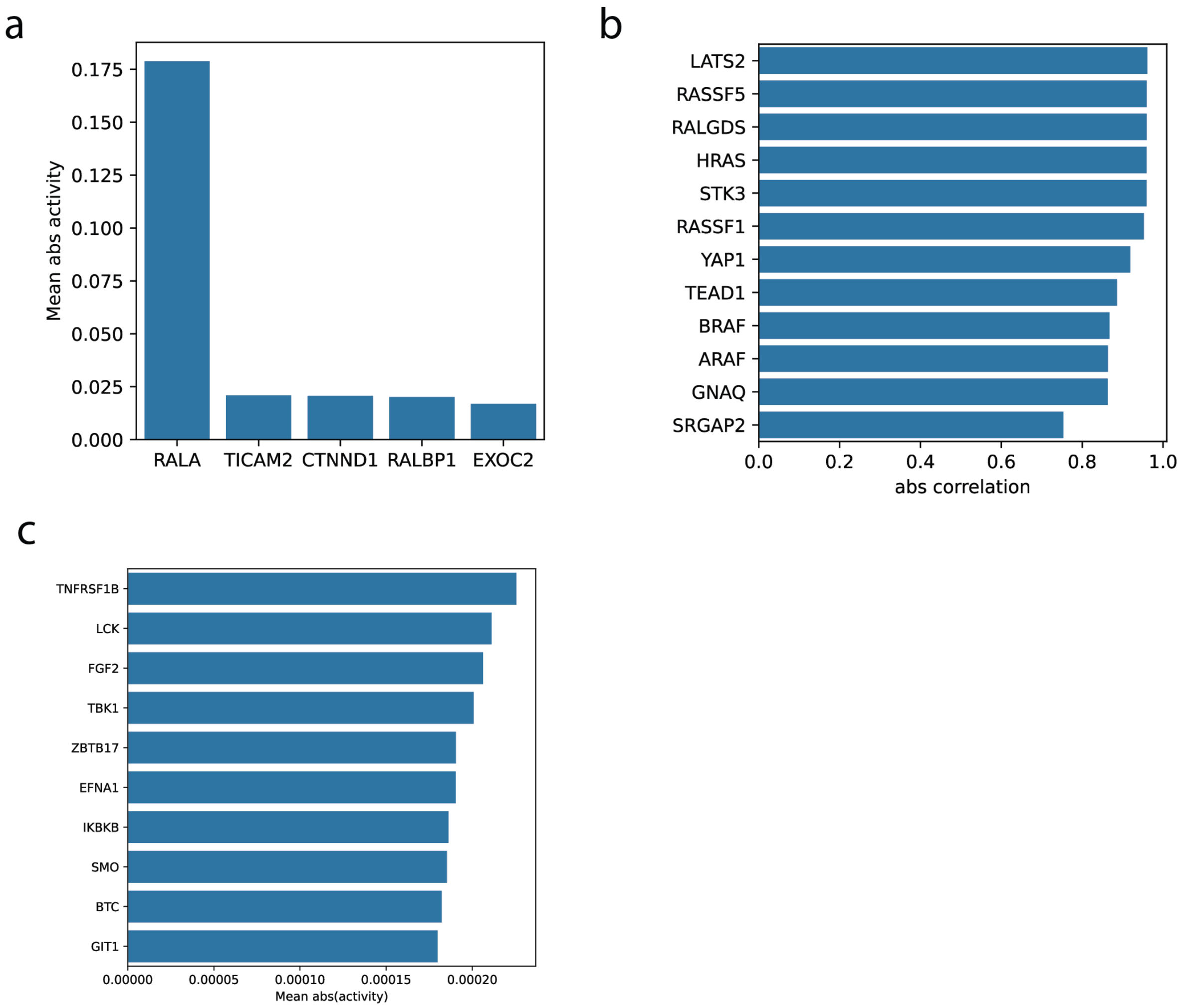

Supplementary Figure 12 Predicting a missing interaction. a model from which the interaction between RALA and RALGDS had purposefully been removed (broken model) was corrected post hoc with by a deep (4 layers) neural network with 200 hidden nodes per layer. The network was trained using the predicted node state of the broken model as input and predicted which nodes should receive an input signal to repair the model, which was evaluated by the fit of the broken model receiving the surrogate signal. **a)** the predicted signals for different nodes. **b)** the most correlated nodes with the surrogate signal, that additionally were unaffected by perturbations in RALA, i.e. not downstream. **c)** predicted surrogate nodes when models trained from scratch in tandem do not include RALA.

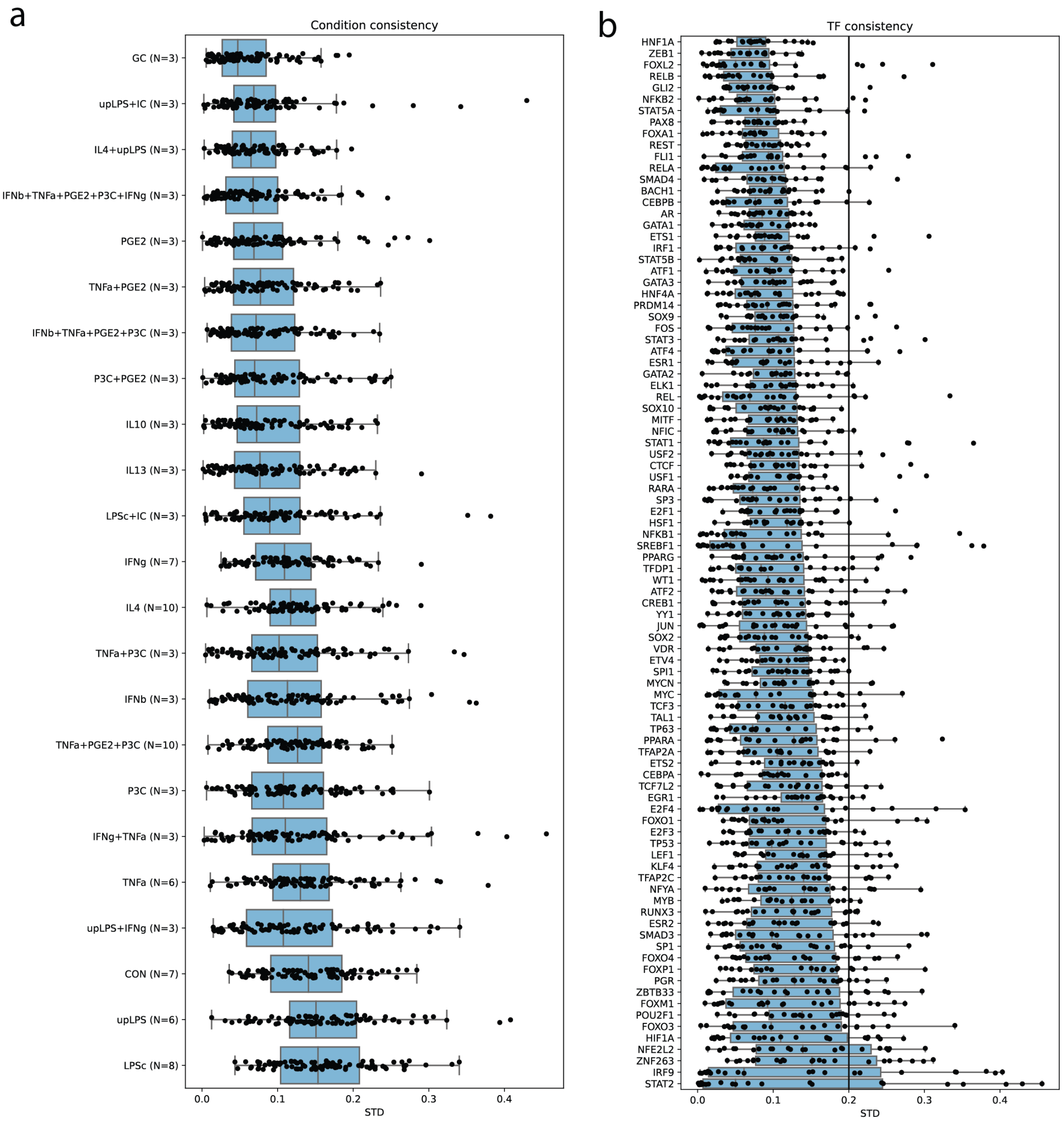

Supplementary Figure 13 TF activities inferred from experimental data from literature. An experimental data set was retrieved from literature[1]. **a)** Standard deviation (STD) of TF activities among biological replicates (number of replicates varies by condition as indicated) by condition (N=23) across TFs (N=92). A cutoff value of 0.2 (black line) for the 75th percentile was selected after inspecting the graph. The standard deviation expected by chance for uniformly distributed values on the interval [0, 1] is 0.29. Four transcription factors did not meet the threshold, STAT2, IRF9, ZNF263 and NFE2L2. **b)** As a, but transcription factors across conditions. The boxes display the median and inter quartile range of the data, whiskers extend to the rest of the data provided that it is within 1.5 inter quartal range of the boundaries of the box.

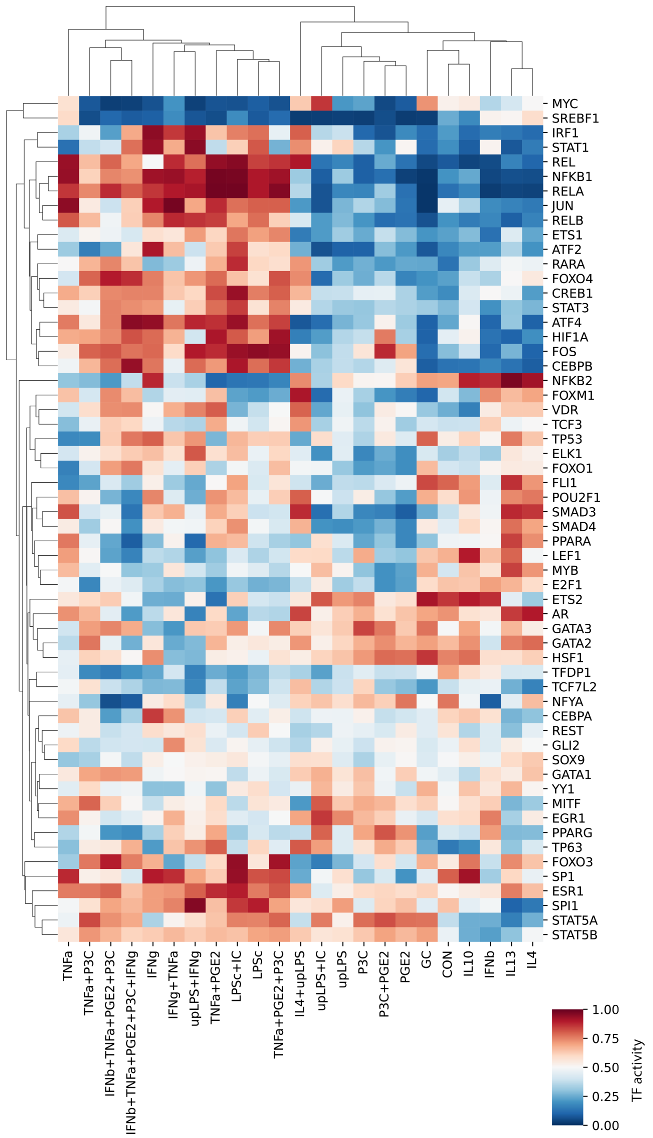

Supplementary Figure 14 Transcription factor activities inferred from literature data. An experimental dataset was retrieved from literature[1] consisting of 23 conditions with combinations of 12 different ligands. Activity was estimated for 58 transcription factors. The inferred activity patterns appear to largely agree with known biology. For example, the transcription factors RelA and RelB are part of the NF-κB signaling cascade and induced by inflammatory ligands, e.g. interferons, lipopolysaccharide (LPS) and TNF[2]. The ligands Il4 and IL13 display similar TF-activity profiles and are opposed to the inflammatory ligands, which is expected since they both signal through IL4R and are known to induce an anti-inflammatory (M2) response[3]. The presence of these ligands is here associated with SMAD3 activity, which may be a secondary effect from secreted TGFβ1[3]. The observed differences in TF-patterns between standard (LPSc) and ultra-pure (upLPS) LPS-qualities, are somewhat unexpected, but may potentially be explained by activation of TLR2 by impurities alongside the expected TLR4 activation[4]. Differences in signaling outcome for these two receptors have previously been noted[2]. Abbreviations: glucocorticoids (GC), ultrapure lipopolysaccharide (upLPS), standard LPS (LPSc), immune complexes (IC), Pam3CSK4 (P3C) and prostaglandin E2 (PGE2).

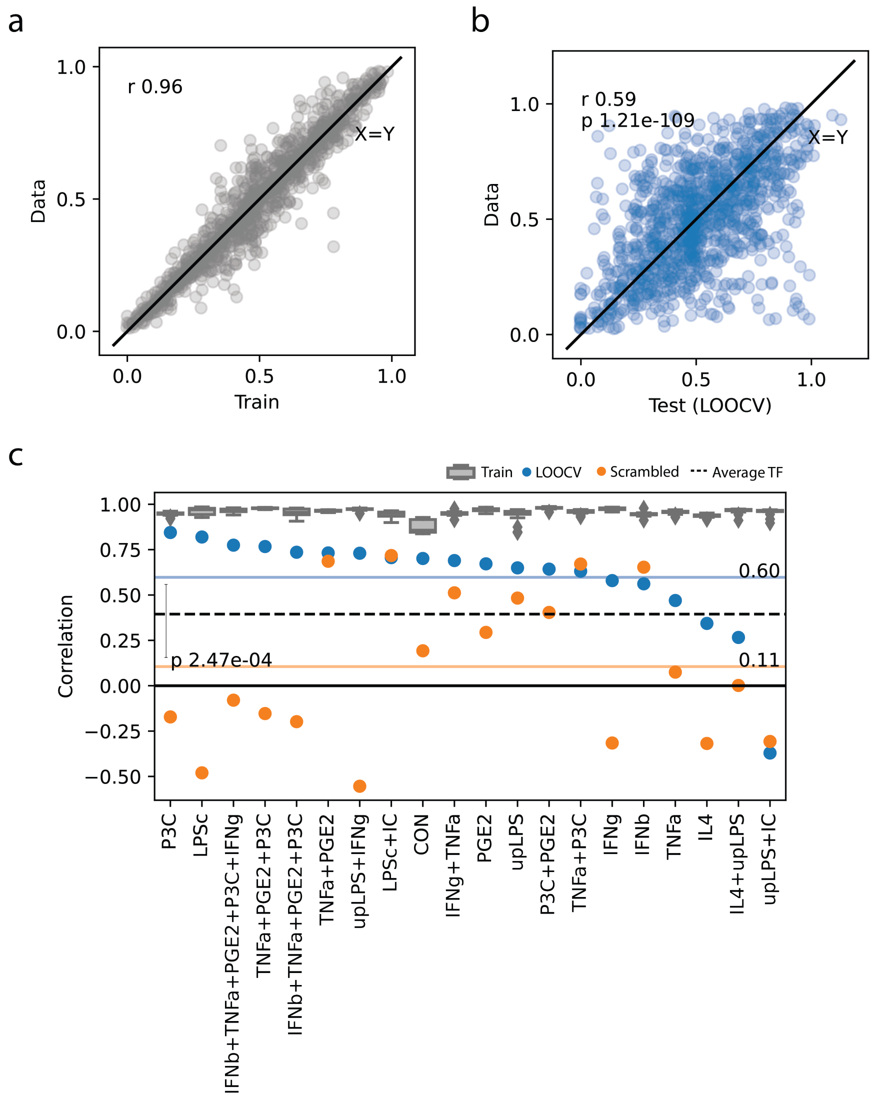

Supplementary Figure 15 Model applied to experimental data from literature. A model was reconstructed to TF activity data inferred from experimental data set was retrieved from literature[1] using the same procedure as for the synthetic network, but also including an immune system specific resource, InnateDB[5] to accommodate the set of ligands used in the experimental study. The list of TFs was restricted to the ones with experimental data available. The model consisted of 1069 signaling nodes and 5886 interactions. **a)** A good fit to the data (r = Pearson correlation) was attained. **b)** Leave one out cross validation (LOOCV) for conditions where all ligands were present in at least two conditions (20 out of 23) showed significant Pearson correlation (r) between prediction and data, the two-tailed p-value for r calculated using scipy.stats.pearsonr. **c)** The correlation within conditions for LOOCV was consistently lower than the train fits, (23 samples x 58 TFs = 1334 datapoints compared to 7000> parameters), but significantly outperformed models trained with scrambled condition labels, statistic calculated using two sided Mann–Whitney U test (n=20). IL4 is among the conditions with poorer generalization, potentially due to confounding interactions with LPS[6]. Abbreviations: glucocorticoids (GC), ultrapure lipopolysaccharide (upLPS), standard LPS (LPSc), immune complexes (IC), Pam3CSK4 (P3C) and prostaglandin E2 (PGE2). The boxes in panel c display the median and inter quartile range of the data, whiskers extend to the rest of the data provided that it is within 1.5 inter quartal range of the boundaries of the box.

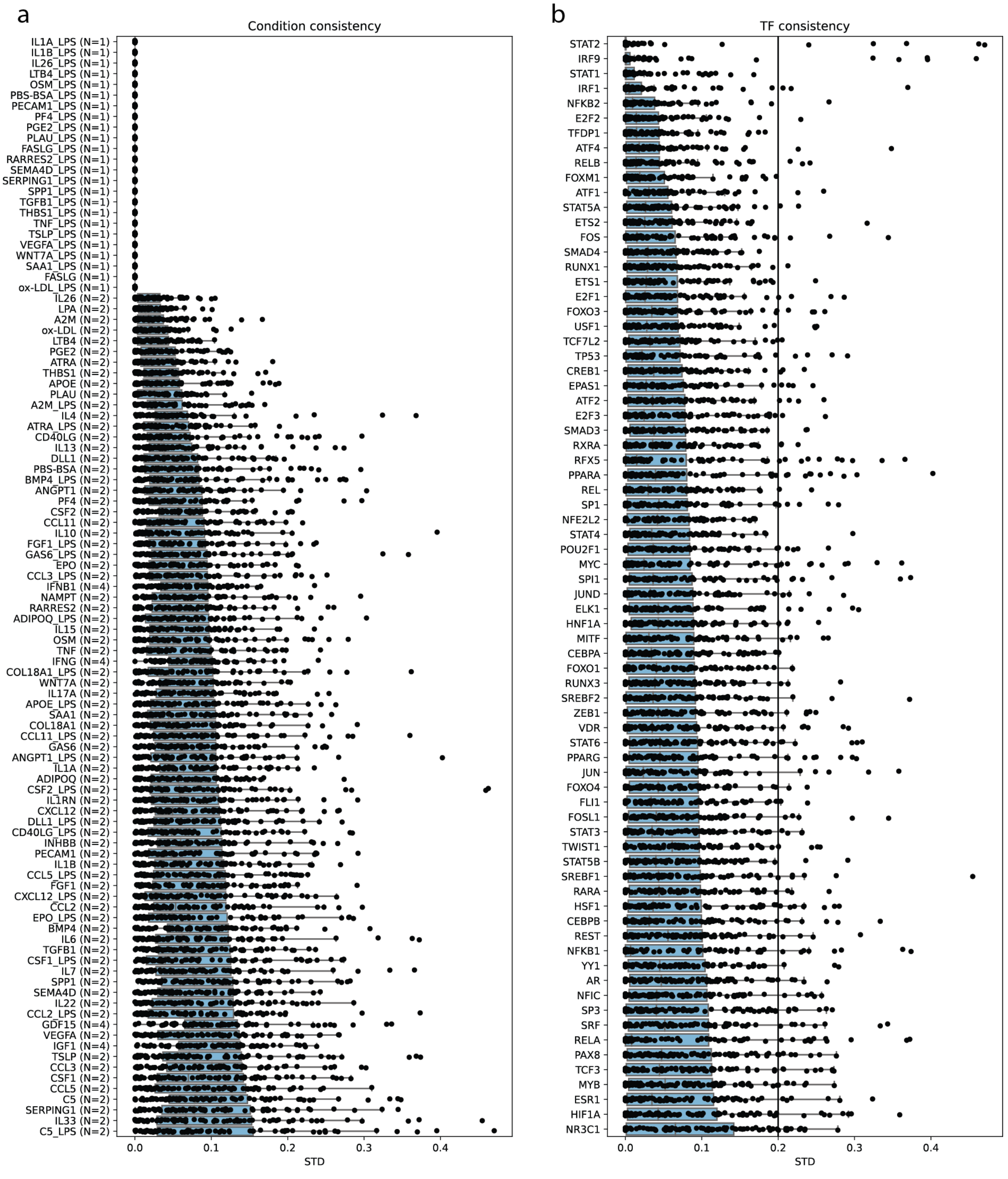

Supplementary Figure 16 TF activities inferred from experimental data. An experimental data set was generated for ligand stimulated macrophages. **a)** Standard deviation (STD) of TF activities among biological replicates (number of replicates varies by condition as indicated, for some conditions there were no replicates, for these the standard deviations is given as 0) by transcription factors (n=74) across conditions (n=103). **b)** As a, but transcription factors across conditions. The boxes display the median and inter quartile range of the data, whiskers extend to the rest of the data provided that it is within 1.5 inter quartal range of the boundaries of the box.

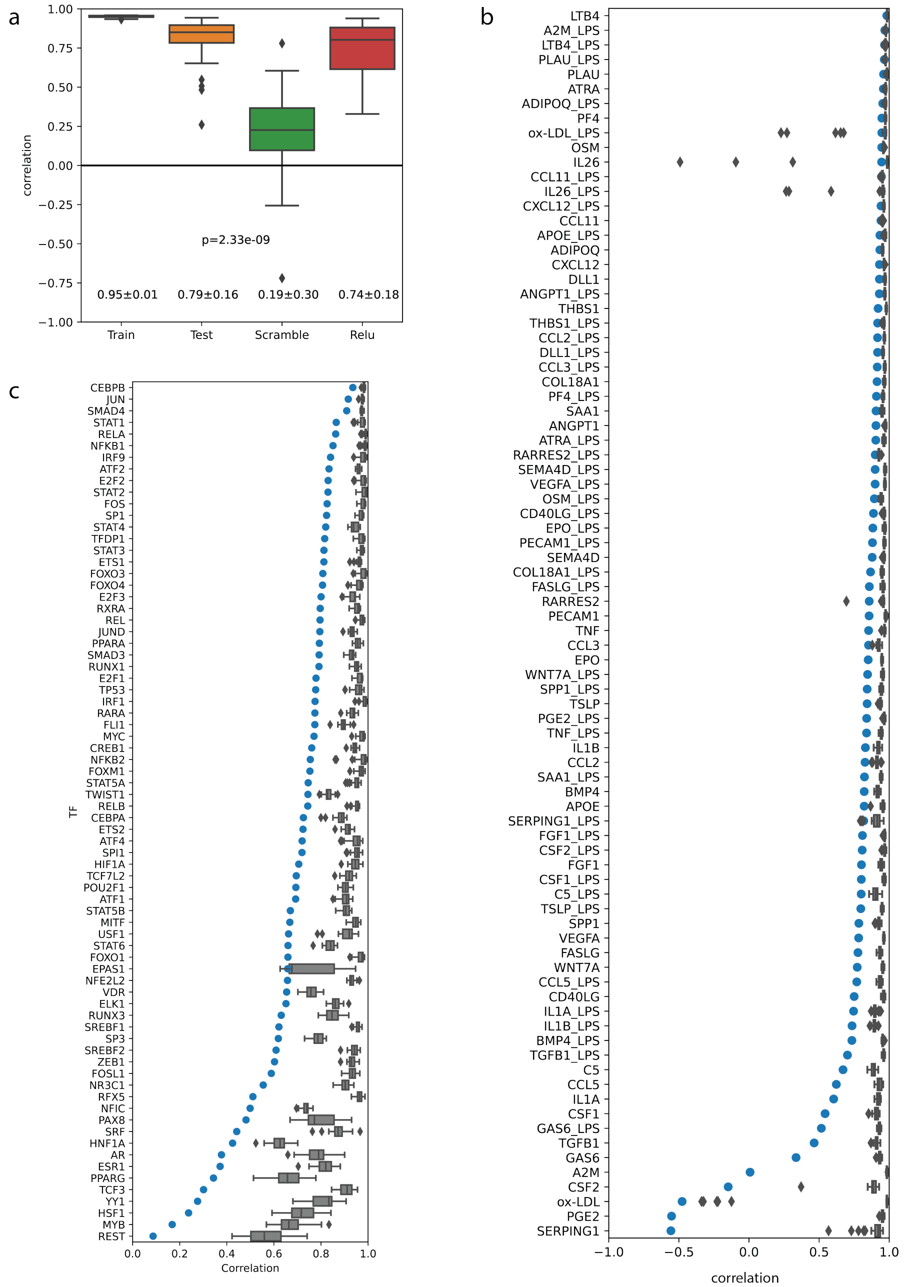

Supplementary Figure 17. Modeling performance for ligand stimulated macrophages. a) cross validation performance for the training and test set, compared with models trained on data in scrambled order, test correlation is significantly higher than scrambled, statistic calculated using two sided Mann–Whitney U test (n=27). A model with the ReLU activation function is included for reference. b) The correlations per TF under the cross validation (blue dots) was consistently lower than the train fits (gray boxplot, n=26) c) The correlation per conditions for the cross validation (blue dots) was consistently lower than the train fits (gray box plot, n=26), and particularly poor for SERPING1, PGE2, ox-LDL and CSF2. Abbreviations of none-gene-name-derived ligands, Leukotriene B4 (LTB4), All-trans retinoic acid (ATRA), Lysophosphatidic acid (LPA), Prostaglandin E2 (PGE2), Lipopolysaccharide (LPS), Oxidized low density lipoprotein (ox-LDL). The boxes display the median and inter quartile range of the data, whiskers extend to the rest of the data provided that it is within 1.5 inter quartal range of the boundaries of the box.

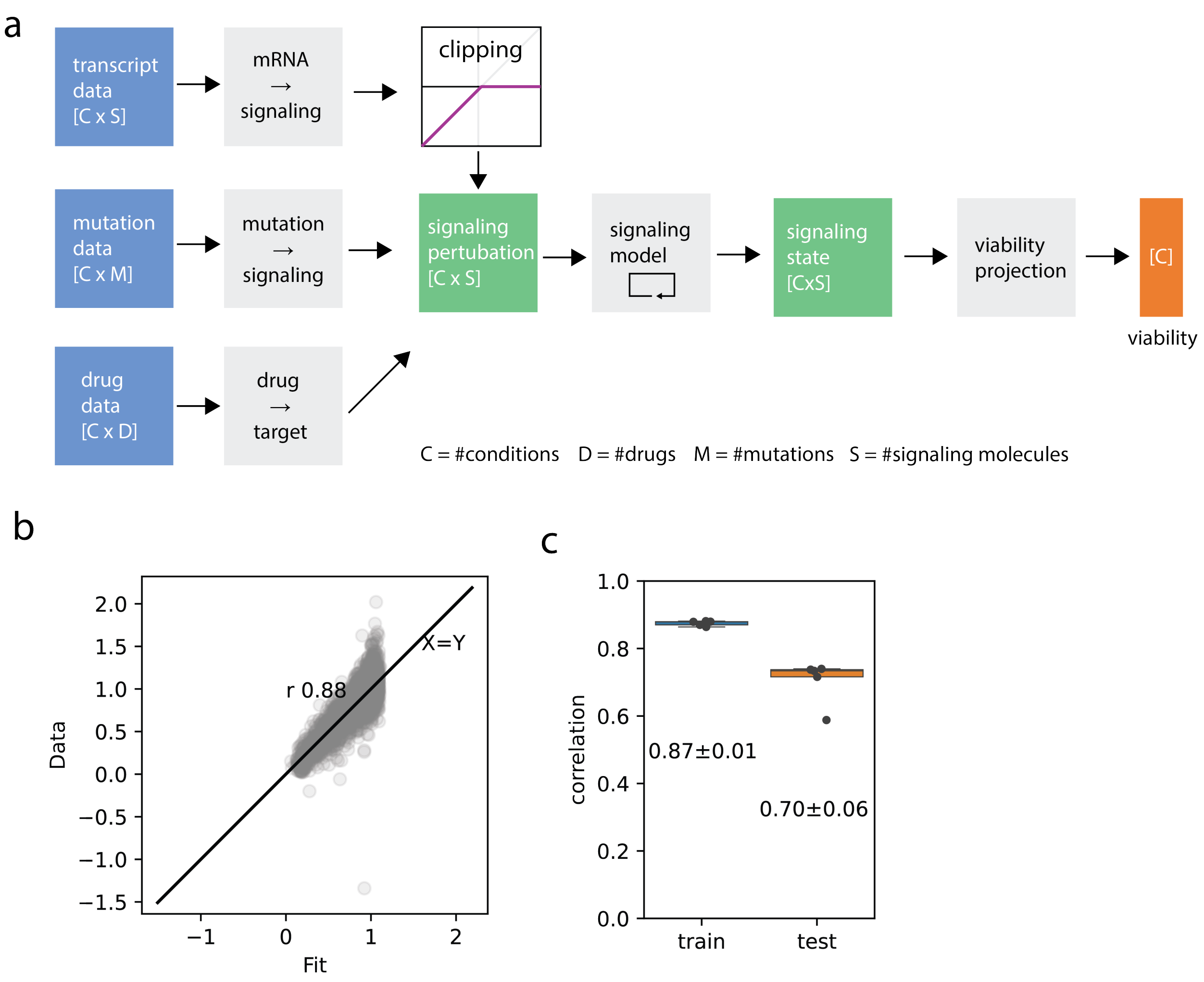

Supplementary Figure 18 Prediction of cell viability in different cancer cell lines in response to drugs. a) The modification of LEMBAS to incorporate as input: (i) cell-line specific basal transcriptomic profiles and (ii) gene mutations in order to predict the cell viability under these types of conditions and (iii) drug concentration. The "mRNA → signaling" layer linearly projects and adds the gene expression of the corresponding signaling protein to the perturbation after applying a non-linear activation function that prevents positive values. The "mutation → signaling" layer projects the targeted/mutated genes of a cell-line into the signaling node space, thus creating a mutation-specific signaling perturbation. The "drug → target" layer utilizes drug-target information, via a trainable drug-target matrix, to predict drug signaling perturbation. Together they form the signaling perturbation which serves as the input of the signaling network model. The output of the recurrent signaling network model is ultimately projected to predict transcription factor activity (internally in the viability projection layer), the weighted sum of which is the predicted cell viability. **b)** Correlation between the viability data and fit by the trained modified LEMBAS model. **c)** Correlation between prediction and data in the training sets and the test sets, using 5-fold cross validation, using the same folds that were used by Fröhlich et al[7].

Supplementary Tables

Supplementary Table 1 Rate parameters for different molecular mechanisms.

	k_1	k_2	k_3	k_4
IA	0.2	0.1	0.2	NA
II	0.2	0.1	0.2	NA
CA	0.5	0.1	NA	NA
CI	0.5	0.1	2	0.1

NA = not applicable

Supplementary Table 2 Queries used to prune the interactions in the OmniPath database

Purpose	Field	Operation	Value
Only human	ncbi_tax_id_target	==	9606
Only OmniPath core set	omnipath	==	True
Ligand-Receptor (LR) interactions	Ligrecextra AND<br>sources	==<br>contains	True<br>‘KEGG’
Signaling interactions	post_translational AND<br>sources	==<br>contains	True<br>‘KEGG’
Regulatory interactions	dorothea AND<br>dorothea_level	==<br>contains	True<br>‘A’ OR ‘B’

Supplementary Table 3, manually defined RL interactions based on uniprot annotation.

Source	Target	Motivation
GC	NR3C1	Glucocorticoid receptor
IC	FCGR3A	Immune complexes may activate FC receptors
	FCGR2A	
IFNb	IFNAR1	Interferon alpha/beta receptor 1&2
	IFNAR2	
IFNg	IFNGR1	Interferon gamma receptor 1&2
	IFNGR2	
IL4	IL4R	Interleukin-4 receptor
IL10	IL10RA	Interleukin-10 receptor subunit alpha
IL13	IL13RA1	Interleukin-13 receptor subunit alpha
P3C	TLR2	P3C activates TLR1 & 2
PGE2	PTGER3	PGE2 activates PTGER1-4
LPSc	TLR2	LPS activates cells through TLR4, the TLR2 activity of LPS-PG is ascribed to a contaminant lipoprotein
	TLR4	
	CD14	CD14 binds bacterial lipopolysaccharide
upLPS	CD14	
	TLR4	Ultrapure LPS only activates TLR4
TNFa	TNFRSF1A	Tumor necrosis factor activates receptor 1 A and B
	TNFRSF1B	

Supplementary Table 4, manually defined RL interactions based on uniprot[8] annotation and literature.

Compound		Receptor	
CHEBI:	Ligand Name	Receptor Name	Id
15647	Leukotriene B4 (LTB4)	Leukotriene B4 receptor 1 (LTB4R)	Q15722
		Leukotriene B4 receptor 2 (LTB4R2)	Q9NPC1
15367	All-trans retinoic acid (ATRA)	Cellular retinoic acid-binding protein 1 (CRABP1)	P29762
		Cellular retinoic acid-binding protein 2 (CRABP2)	P29373
32957	Lysophosphatidic acid (LPA)	Lysophosphatidic acid receptor 1 (LPAR1)	Q92633
		Lysophosphatidic acid receptor 2 (LPAR2)	Q9HBW0
		Lysophosphatidic acid receptor 3 (LPAR3)	Q9UBY5
		Lysophosphatidic acid receptor 4 (LPAR4)	Q99677
		Lysophosphatidic acid receptor 5 (LPAR5)	Q9H1C0
		Lysophosphatidic acid receptor 6 (LPAR6)	P43657
15551	Prostaglandin E2 (PGE2)	Prostaglandin E2 receptor EP1 subtype (PTGER1)	P34995
		Prostaglandin E2 receptor EP2 subtype (PTGER2)	P43116
		Prostaglandin E2 receptor EP3 subtype (PTGER3)	P43115
		Prostaglandin E2 receptor EP4 subtype (PTGER4)	P35408
16412	Lipopolysaccharide (LPS)	Toll-like receptor 4 (TLR4)	O00206
		Toll-like receptor 2 (TLR2)	O60603
		Lipopolysaccharide-binding protein (LBP)	P18428
		Monocyte differentiation antigen CD14 (CD14)	P08571
		Lymphocyte antigen 96 (LY96)	Q9Y6Y9
60151	Oxidized low density lipoprotein (ox-LDL)	Oxidized low-density lipoprotein receptor 1 (OLR1)[9]	P78380
		Platelet glycoprotein 4 (CD36)[9]	P16671
		Macrophage scavenger receptor types I and II (MSR1)[9]	P21757
		Stabilin-1 (STAB1) [9]	Q9NY15
		C-X-C motif chemokine 16 (CXCL16)[9]	Q9H2A7
		Advanced glycosylation end product-specific receptor (AGER)[9]	Q15109
		Monocyte differentiation antigen CD14 (CD14)[10].	P08571

Supplementary Note 1

Derivation of backpropagation at steady state. Let the state at time step n be defined as,

$$\mathbf{z}_n = \mathbf{A}\mathbf{h}_{n-1} + \mathbf{b}$$

$$\mathbf{h}_n = \sigma(\mathbf{z}_n)$$

$$\mathbf{y}_n = \mathbf{p}\mathbf{x}_n.$$

Apply the chain rule to some loss function $L = (\mathbf{y}_n)$,

$$\frac{dL}{\mathbf{z}_n} = \frac{dL}{d\mathbf{h}_n}\frac{d\mathbf{h}_n}{d\mathbf{z}_n} = \frac{dL}{d\mathbf{h}_n}\ \sigma'(\mathbf{z}_n).$$

where

$$\frac{dL}{d\mathbf{h}_n} = \frac{dL}{d\mathbf{y}_n}\frac{d\mathbf{y}_n}{d\mathbf{x}_n} + \frac{dL}{d\mathbf{z}_{n+1}}\frac{d\mathbf{z}_{n+1}}{d\mathbf{h}_n} = \frac{dL}{d\mathbf{y}_n}\frac{d\mathbf{y}_n}{d\mathbf{h}_n} + \frac{dL}{d\mathbf{z}_{n+1}}\mathbf{A}$$

Combining the two equations we get the following recursive formula

$$\frac{dL}{d\mathbf{z}_n} = \left(\frac{dL}{d\mathbf{y}_n}\frac{d\mathbf{y}_n}{d\mathbf{h}_n} + \frac{dL}{d\mathbf{z}_{n+1}}A\right)\ \sigma'(\mathbf{z}_n).$$

If we assume steady state,

$$\mathbf{z}_{\mathrm{ss}} = \mathbf{A}\mathbf{h}_{\mathrm{ss}} + \mathbf{b}$$

we get the following linear equation system

$$\frac{dL}{d\mathbf{z}_{\mathrm{ss}}} = \left(\frac{dL}{d\mathbf{y}_{\mathrm{ss}}}\frac{d\mathbf{y}_{\mathrm{ss}}}{d\mathbf{h}_{\mathrm{ss}}} + \frac{dL}{d\mathbf{z}_{\mathrm{ss}}}\mathbf{A}\right)\ \sigma'(\mathbf{z}_{\mathrm{ss}})$$

expressed in gradient form

$$\nabla\mathbf{z}_{\mathrm{ss}} = \sigma'(\mathbf{z}_{\mathrm{ss}})\odot\left(\mathbf{A}^{\mathrm{T}}\nabla\mathbf{z}_{\mathrm{ss}} + \nabla L\ \right).$$

This equation can be solved by iteration from some initial guess for $\nabla\mathbf{z}_n$ (e.g. $\nabla\mathbf{z}_n = 0$. From this the gradients of the parameters A and b can be calculated with respect to L as

$$\frac{dL}{d\mathbf{A}} = \frac{dL}{d\mathbf{z}_{\mathrm{ss}}}\frac{d\mathbf{z}_{\mathrm{ss}}}{d\mathbf{A}} = \mathbf{h}_{\mathrm{ss}}\nabla\mathbf{z}_{\mathrm{ss}}^{\mathrm{T}}.$$

$$\frac{dL}{d\mathbf{b}} = \frac{dL}{d\mathbf{z}_{\mathrm{ss}}}\frac{d\mathbf{z}_{\mathrm{ss}}}{d\boldsymbol{b}} = \mathbf{1}\cdot\nabla\mathbf{z}_{\mathrm{ss}}.$$

Supplementary Note 2

Derivation of backpropagation without assuming steady state in advance. If we prefer to not assume steady state at this stage, we can note that the loss function is evaluated at a specific timestep (k) and is zero elsewhere. The loss depends on states from timestep 1 to k and the loss is zero with respect to timesteps outside of this range.

$$\frac{dL}{d\mathbf{y}_k}\frac{d\mathbf{y}_k}{d\mathbf{h}_k} = \nabla L, \frac{dL}{d\mathbf{z}_0} = 0, \frac{dL}{d\mathbf{z}_{k+1}} = 0, \nabla\mathbf{z}_k = \sigma'(\mathbf{z}_k)\odot(\nabla L)$$

With these boundary conditions the loss can be evaluated as

$$\nabla\mathbf{z}_n = \sigma'(\mathbf{z}_n)\odot\left(\mathbf{A}^{\mathrm{T}}\nabla\mathbf{z}_{n+1}\right) = \left(\prod_n^k \sigma'(\mathbf{z}_n)\odot\mathbf{A}^{\mathrm{T}}\right)\nabla L$$

Since parameters are the same across all time steps, their gradients can be summed into a quantity that is independent of the time step:

$$\frac{dL}{d\mathbf{A}} = \sum_1^k \nabla\mathbf{z}_i\,\mathbf{h}_i = \sum_1^k\left(\prod_i^k \sigma'(\mathbf{z}_i)\odot\mathbf{A}^{\mathrm{T}}\right)\nabla L\mathbf{h}_i$$

$$\frac{dL}{d\mathbf{b}} = \sum_1^k \nabla\mathbf{z}_i\cdot\mathbf{1} = \sum_1^k\left(\prod_i^k \sigma'(\mathbf{z}_i)\odot\mathbf{A}^{\mathrm{T}}\right)\nabla L\cdot\mathbf{1}.$$

Note that the conserved part of these equations can be written in recursive form as

$$\mathbf{s}_n = \sigma'(\mathbf{z}_{k+1-n})\odot\mathbf{A}^{\mathrm{T}}(\mathbf{s}_{n-1} + \nabla L).$$

For large n the repeated multiplication will cause later terms to vanish, and the recursion can be truncated at the step (t) where this occurs. If forward propagation has reached steady state all of the non-truncated values will be from the steady state (and if not, then a larger k can be chosen, see next section for further analysis on the requirements for steady state to occur). We can then simplify the equation

$$\mathbf{s}_t = \boldsymbol{\sigma}'(\mathbf{z}_{\mathrm{ss}})\odot\mathbf{A}^{\mathrm{T}}(\mathbf{s}_t + \nabla L).$$

This has the same form as the steady state expression derived above, and the same analysis for parameter gradients applies.

Supplementary Note 3

Derivation, spectral radius determines rate of convergence. Let the state at time step n be defined as,

$$\mathbf{h}_n = \sigma(\mathbf{A}\mathbf{h}_{n-1} + \mathbf{b})$$

then a steady state $\mathbf{h}_{\mathrm{ss}}$ is defined as

$$\mathbf{h}_{\mathrm{ss}} = \sigma(\mathbf{A}\mathbf{h}_{\mathrm{ss}} + \mathbf{b})$$

Taylor expand to the first order around the steady state

$$\mathbf{h}_n = \sigma(\mathbf{A}\mathbf{x}_{\mathrm{ss}} + \mathbf{b}) + \left(\mathbf{I}\sigma'(\mathbf{A}\mathbf{h}_{\mathrm{ss}} + \mathbf{b})\right) \odot \left((\mathbf{A}\mathbf{h}_{n-1} + \mathbf{b}) - (\mathbf{A}\mathbf{h}_{\mathrm{ss}} + \mathbf{b})\right)$$
$$= \mathbf{h}_{ss} + \left(\mathbf{I}\sigma'(\mathbf{A}\mathbf{h}_{\mathrm{ss}} + \mathbf{b})\right) \odot \left(\mathbf{A}(\mathbf{h}_{n-1} - \mathbf{h}_{\mathrm{ss}})\right),$$

where $\odot$ is element wise multiplication and I the identity matrix. Let a displacement from the steady state at time step n be defined as

$$\Delta\mathbf{h}_n = \mathbf{h}_n - \mathbf{h}_{\mathrm{ss}},$$

and let T be defined as

$$T = \left(\mathbf{I}\sigma'(\mathbf{A}\mathbf{h}_{\mathrm{ss}} + \mathbf{b})\right) \odot \mathbf{A},$$

we can then rewrite the equation as

$$\Delta\mathbf{h}_n = \mathbf{T}\Delta\mathbf{h}_{n-1}.$$

By repeated insertion we have

$$\Delta\mathbf{h}_n = \mathbf{T}^n\Delta\mathbf{h}_0.$$

For an initial non-zero displacement from steady state $\Delta\mathbf{h}_0$ to reach $\mathbf{h}_{ss}$ at time n,

$$0 \approx \mathbf{T}^n(\Delta\mathbf{h}_0),$$

this requires that

$$\mathbf{T}^n \approx 0,\ \rho(\mathrm{T}) < 1,$$

where $\rho(\mathrm{T})$ is the eigenvalue of T with largest absolute value. An approximation of the number of time steps (n) required depends on the desired precision and is given as

$$n \propto \frac{\log([\text{precision}])}{\log(\rho(\mathbf{T}))}.$$

To impose the spectral radius as a soft constraint, several forms for the regularization function were considered, including a reciprocal and exponential dependence on the spectral radius. A reciprocal function, while perhaps the most intuitive, undergoes a singularity at a spectral radius of 1 with infinite derivative, which is numerically impractical for gradient decent. An exponential function with parameters (a and k) could be fitted to behave similarly to the reciprocal function, but without singularity issues.

$$L_\rho = a\left(e^{(k\rho)} - 1\right)$$

Supplementary Note 4

Analysis of the framework's algorithmic complexity. The computation of state vectors relies on sparse matrix multiplications that increases linearly with the number of interactions (z, number of elements in matrix) and squared by the number of signaling nodes (n, width/height of matrix). Because the number of interactions is expected to increase as a function of the number of nodes, the worst-case time complexity becomes n^3, corresponding to dense matrix multiplication. The calculation of spectral radius and eigen vectors of sparse matrixes used for regularization is naively of complexity n^3, but is calculated using a stochastic algorithm with a complexity n^2 and a factor that depends on the size gap[11] between the eigen values (g). This eigengap can be expected to be a function of n since the total number of eigen values (n) are constrained to a disk with finite area ($1^2\pi = \pi$). The model is iteratively evaluated until steady state, and computation time increases linearly with the number of time steps (t). Through matrix operations, different conditions can be evaluated in parallel, but the algorithmic complexity depends linearly on the number of conditions, that are typically divided into mini-batches (b) of some size (r). To train a generalizable model, the forward pass must be executed multiple times and each forward pass is accompanied by a backward pass with the same complexity. The number of epochs (e) required for the training to converge may depend on n and the number of conditions may in practice also depend weakly on n, as more parameters could be expected to require more data. With this notation the overall complexity depends on many factors,

$$\mathrm{O}\big(er(btzn^2 + gn^2)\big),$$

an expression with explicit squared complexity with respect to n. However, due to relations between n and some of the other parameters, the complexity can be expected to be worse in practice.

Supplementary Note 5

The algorithm presented below is a manual autograd function and thus requires both a forward call and backward call (for the partial derivative). To accommodate dot multiplication, data must be transposed to [features x samples], as compared to the machine learning standard of [samples x features]. In practice this is performed by the algorithm but for the sake of this presentation it is assumed to already have occurred.

Sparse one-to-one RNN (forward)

[x_{ss}, x_{raw}] = **function** forward(b_{in} weights, bias, A)

Input:

b_{in}, [n x s] matrix for s samples and n state variables, zero-padded with ligand concentrations at their corresponding positions.

weights, [i x 1] vector containing the non-zero elements.

bias, [n x 1] vector of biases for each state variable.

A, [n x n] sparse matrix with i non-zero elements that describes the network structure.

Output:

x_{ss} [n x s] vector of predicted steady states.

x_{raw} [n x s] vector of predicted steady states before applying activation function.

A.nze = weights *#loads the weights into their correct positions in the sparse matrix A*

b = b_{in} + bias

x_{ss} = zeros [n x s] #initiate x_{ss} as an all zero matrix with dimensions n x s

for max_iter **steps**: *#Max_iter is set to 150 in this study*

x_{ss}= **dot product**(A, x_{ss})

x_{ss}= x_{ss}**+** b *#vector b is repeated for each sample in x_{ss}(broadcasted)*

x_{ss}= **activation**(x_{ss}) *#element-wise activation function, specified above*

x_{raw} **= dot product**(A, x_{ss}) **+** b *#When x_{ss} is at steady state, this is same as inv(activation(x_{ss}))*

Sparse one-to-one RNN (backward)

[z, dw, db] **= function** backward(err, x_{ss}, x_{raw}, A)

Input:

err, [n x d] matrix containing gradient back propagated from comparing TF activities projected from x_{ss} to data.

x_{ss}, [n x d] matrix see forward.

x_{raw}, [n x s] matrix see forward.

A, [n x n] matrix see forward.

Output:

z, [n x s] error to be back propagated to proceeding layers.

dw, [i x 1] gradient of the weight vector.

db, [n x 1] gradient of the bias vector.

xDelta **= activation'(**x_{raw}**)** *#the derivative of the activation function*

T = **transpose**(A) #targets and sources are reversed for backward pass

z = An all zero matrix with same dimensions as err

for max_iter steps:

z = **dot product**(T, z) + err

z = **element-wise product**(xDelta, z)

z = **clipping**(z) *#gradient clipping as described below*

db = **rowsum**(z)

dw = **rowsum**(**element-wise product**(x_{ss}[source], z[target])) *# as further explained below*

For calculation of the weight gradients, only the non-zero elements of A are relevant. Therefore, the complete dot multiplication between the matrixes x_{ss} and grad can be replaced by elementwise multiplication and subsequent summation for the elements corresponding to the source (column) and target (row) of each weight.

To prevent clipping under normal conditions, the clipping function is constructed with a linear segment between two saturating tanh regions, resulting in a continuous and monotonically increasing function from -2 to 2,

$$\begin{array}{cc} & \text{clipping}(z) \\ z \leq -1 & \tanh(z+1)-1 \\ -1 < z \leq 1 & z \\ 1 < z & \tanh(z-1)+1 \end{array}.$$

Supplementary Note 6

The algorithm presented below is a manual autograd function and thus requires both a forward call and backward call (for the partial derivative).

Spectral radius (forward)

[ρ, e, v] = **function** forward(**A, weights**)

Input:

A, [n x n] sparse matrix with i non-zero elements that describes the network structure.

weights, [i x 1] vector of linearized non-zero weights of **A**

Output:

ρ, scalar, the spectral radius

e, complex scalar, the eigen value of **A** with largest absolute value

$\mathbf{v}$, [n x 1] complex vector, the right eigen vector of **A**

T = A *#Create T with same structure as A*

T.nze = $\mathbf{weights}$ *#load the linearized weights into correct positions in the sparse matrix T*

e, v **= eigs**(T) *#returns the eigenvalue (e) with largest absolute value and its right eigenvector (v)*

ρ=**abs**(e)

Spectral radius (backward)

[dw] **= function** backward(grad, v, e)

Input:

grad, a scalar with the backpropagated gradient of the barrier function

v, [n x 1] complex vector, see forward

e, complex scalar, see forward

Output:

dw gradient of the weight vector

e, w = **eigs**(**tanspose**(T), e) *#gives the eigen vector* w *closest to the eigenvalue to e (the left eigen vector)*

divisor = **dot product**(**transpose**(w), v)

direction = e/**abs**(e)

delta = **elementwise product** (w[target], v[source])/divisor

delta = **real**(delta/direction)

delta = **limit norm**(delta, 10) # optionally the norm of delta can be constrained to a finite value

dw = grad * delta

Since eigs is a stochastic algorithm it sometimes, under rare circumstances, fails to return eigen values before timing out, then zeros are returned as gradient.

Supplementary References

1. Xue, J. *et al.* Transcriptome-based network analysis reveals a spectrum model of human macrophage activation. *Immunity* **40**, 274–288 (2014).
2. Dorrington, M. G. & Fraser, I. D. C. NF-κB signaling in macrophages: dynamics, crosstalk, and signal integration. *Frontiers in Immunology* vol. 10 705 (2019).
3. Borthwick, L. A. & Wynn, T. A. IL-13 and TGF-β1: core mediators of fibrosis. *Curr. Pathobiol. Rep.* **3**, 273–282 (2015).
4. Liu, J. *et al.* Molecular Mechanism of the Bifunctional Role of Lipopolysaccharide in Osteoclastogenesis. *J. Biol. Chem.* **284**, 12512–12523 (2009).
5. Breuer, K. *et al.* InnateDB: systems biology of innate immunity and beyond—recent updates and continuing curation. *Nucleic Acids Res.* **41**, D1228–D1233 (2013).
6. Major, J., Fletcher, J. E. & Hamilton, T. A. IL-4 pretreatment selectively enhances cytokine and chemokine production in lipopolysaccharide-stimulated mouse peritoneal macrophages. *J. Immunol.* **168**, 2456 LP – 2463 (2002).
7. Fröhlich, F. *et al.* Efficient parameter estimation enables the prediction of drug response using a mechanistic pan-cancer pathway model. *Cell Syst.* **7**, 567-579.e6 (2018).
8. Bateman, A. *et al.* UniProt: The universal protein knowledgebase. *Nucleic Acids Res.* **45**, D158–D169 (2017).
9. Cuthbert, G. A., Shaik, F., Harrison, M. A., Ponnambalam, S. & Homer-Vanniasinkam, S. Scavenger Receptors as Biomarkers and Therapeutic Targets in Cardiovascular Disease. *Cells* vol. 9 (2020).
10. Miller, Y. I. *et al.* Minimally Modified LDL Binds to CD14, Induces Macrophage Spreading via TLR4/MD-2, and Inhibits Phagocytosis of Apoptotic Cells *. *J. Biol. Chem.* **278**, 1561–1568 (2003).
11. Morgan, R. On restarting the Arnoldi method for large nonsymmetric eigenvalue problems. *Math. Comput.* **65**, 1213–1230 (1996).
