# Supplementary material for: Artificial neural networks enable genome-scale simulations of intracellular signaling
Source: Nat Commun. 2022 Jun 2;13:3069. doi: 10.1038/s41467-022-30684-y (PMC9163072; doi:10.1038/s41467-022-30684-y)
Supplement: Supplementary file 2 — Description of Additional Supplementary Information [file 41467_2022_30684_MOESM2_ESM.docx]

# Inventory of supporting information

# Supplementary Data1

List of manually curated interactions.

# Source data

Source data for the figures.

# Supplementary Information

Containins supplementary figures 1-18, supplementary tables 1-4, and supplementary notes 1-6.

## Supplementary Figure 1

Fitting ODEs with a Michaelis Menten like (MML) activation function.

## Supplementary Figure 2

Numerical comparison of auto-grad and steady-state for a random network.

## Supplementary Figure 3

Alternative methods for solving the forward and backward pass.

## Supplementary Figure 4

Adversarial test data and network structure.

## Supplementary Figure 5

Assuming steady state in a dynamical model.

## Supplementary Figure 6

Derivative of spectral radius.

## Supplementary Figure 7

Variability in output depending on input for random and biological networks.

## Supplementary Figure 8

Automatic parameterization of the small signaling network.

## Supplementary Figure 9

Principal component analysis of synthetic model output.

## Supplementary Figure 10

Empirical tests of time complexity

## Supplementary Figure 11

Training performance on a synthetic data.

## Supplementary Figure 12

Predicting missing interactions.

## Supplementary Figure 13

TF activities inferred from experimental data from literature.

## Supplementary Figure 14

Transcription factor activities inferred from literature data.

## Supplementary Figure 15

Model applied to experimental data from literature.

## Supplementary Figure 16

TF activities inferred from experimental data.

## Supplementary Figure 17

Modeling performance for ligand stimulated macrophages.

## Supplementary Figure 18

Prediction of cell viability in different cancer cell lines in response to drugs.

## Supplementary Table 1

Manually assigned rate parameters for the ODE models of different molecular mechanisms.

## Supplementary Table 2

Queries used to prune the interactions in the OmniPath database.

## Supplementary Table 3

Manually defined receptor ligand interactions for the macrophage model for literature data.

## Supplementary Table 4

Manually defined receptor ligand interactions for the macrophage model for the ligand stimulated macrophages.

## Supplementary Note 1

Derivation of the partial gradient of backpropagation at steady state.

## Supplementary Note 2

Derivation of the partial gradient using backpropagation without assuming steady state in advance.

## Supplementary Note 3

Derivation of the rate on convergence dependency on the spectral radius.

## Supplementary Note 4

Analysis of the framework’s algorithmic complexity.

## Supplementary Note 5

Recurrent neural network algorithm.

## Supplementary Note 6

Spectral radius constraint algorithm.
