# Supplementary material for: Artificial neural networks enable genome-scale simulations of intracellular signaling
Source: Nat Commun. 2022 Jun 2;13:3069. doi: 10.1038/s41467-022-30684-y (PMC9163072; doi:10.1038/s41467-022-30684-y)
Supplement: Supplementary file 4 — Reporting Summary [file 41467_2022_30684_MOESM4_ESM.pdf]

nature portfolio

Corresponding author(s): Douglas A. Lauffenburger

Last updated by author(s): Apr 29, 2022

Reporting Summary

Nature Portfolio wishes to improve the reproducibility of the work that we publish. This form provides structure for consistency and transparency in reporting. For further information on Nature Portfolio policies, see our Editorial Policies and the Editorial Policy Checklist.

Statistics

For all statistical analyses, confirm that the following items are present in the figure legend, table legend, main text, or Methods section.

n/a	Confirmed	
[ ]	[x]	The exact sample size (*n*) for each experimental group/condition, given as a discrete number and unit of measurement
[x]	[ ]	A statement on whether measurements were taken from distinct samples or whether the same sample was measured repeatedly
[ ]	[x]	The statistical test(s) used AND whether they are one- or two-sided<br>*Only common tests should be described solely by name; describe more complex techniques in the Methods section.*
[x]	[ ]	A description of all covariates tested
[x]	[ ]	A description of any assumptions or corrections, such as tests of normality and adjustment for multiple comparisons
[ ]	[x]	A full description of the statistical parameters including central tendency (e.g. means) or other basic estimates (e.g. regression coefficient) AND variation (e.g. standard deviation) or associated estimates of uncertainty (e.g. confidence intervals)
[ ]	[x]	For null hypothesis testing, the test statistic (e.g. *F*, *t*, *r*) with confidence intervals, effect sizes, degrees of freedom and *P* value noted<br>*Give P values as exact values whenever suitable.*
[x]	[ ]	For Bayesian analysis, information on the choice of priors and Markov chain Monte Carlo settings
[x]	[ ]	For hierarchical and complex designs, identification of the appropriate level for tests and full reporting of outcomes
[ ]	[x]	Estimates of effect sizes (e.g. Cohen's *d*, Pearson's *r*), indicating how they were calculated

Our web collection on statistics for biologists contains articles on many of the points above.

Software and code

Policy information about availability of computer code

Data collection

Some of the data used was retrieved from public repositories using their web interface.
A script to download the data (downloadLiteratureData.py) is provided at github.com/Lauffenburger-Lab/LEMBAS

Data analysis	Custom code available at github.com/Lauffenburger-Lab/LEMBAS, doi.org/10.5281/zenodo.6532706.<br>Relying on the following software and packages<br>python 3.7.10<br>-matplotlib 3.3.4<br>-networkx 2.5 (required for network reconstruction)<br>-numpy 1.20.2<br>-pandas 1.1.3<br>-pytorch 1.6.0<br>-scipy 1.6.2<br>-seaborn 0.11.0<br><br>MATLAB R_2020_a (required for assembly of transcriptomics data from literature, network visualization and complexity tests)<br><br>R 4.0.3 (required for TF activity estimates)<br>-dorothea 1.0.1<br>-DESeq2 1.28.1<br>-limma_3.44.3

For manuscripts utilizing custom algorithms or software that are central to the research but not yet described in published literature, software must be made available to editors and reviewers. We strongly encourage code deposition in a community repository (e.g. GitHub). See the Nature Portfolio guidelines for submitting code & software for further information.

Data

Policy information about availability of data

All manuscripts must include a data availability statement. This statement should provide the following information, where applicable:

- Accession codes, unique identifiers, or web links for publicly available datasets
- A description of any restrictions on data availability
- For clinical datasets or third party data, please ensure that the statement adheres to our policy

The count matrices for the 60-ligand RNA-sequencing dataset generated in this study have been deposited in the in the Gene Expression Omnibus under accession number GSE202515.
The OmniPath interaction database used in this study is available through their web site (archive.omnipathdb.org/omnipath_webservice_interactions__20210621-20211113.tsv.xz).
The transcriptomics data for ligand stimulated macrophages from Xue et al 20144 used in this study is available at ArrayExpress under accession code E-GEOD-46903 (ebi.ac.uk/arrayexpress/experiments/E-GEOD-46903).
The processed cell viability data and cell line mutation profile from Fröhlich et al 201848 used in this study is available on Zenodo (doi.org/10.5281/zenodo.1472794).
The associated basal cell line expression data (RPKM) used in this study is available at the depmap portal under CCLE 2019 (depmap.org/portal/download).

Field-specific reporting

Please select the one below that is the best fit for your research. If you are not sure, read the appropriate sections before making your selection.

☒ Life sciences ☐ Behavioural & social sciences ☐ Ecological, evolutionary & environmental sciences

For a reference copy of the document with all sections, see nature.com/documents/nr-reporting-summary-flat.pdf

Life sciences study design

All studies must disclose on these points even when the disclosure is negative.

Sample size	No statistical method was used to predetermine sample size, however sample size requirements were estimated in this study by simulations on synthetic data (Fig. 5a). These indicated that 100 experimental conditions could be expected to yield a predictive performance (Pearson correlation) of around r=0.8 on test data.
Data exclusions	Samples were removed based on quality criteria, i.e. if number of measured transcripts <2e6 or number of measured genes per sample <5000.
Replication	No attempts were made to reproduce the transcriptomics data.
Randomization	The experiments were not randomized. The samples were purchased from the Massachusetts General Hospital (MGH) Blood Donor Center and received without any identifying information (sex, age, etc). The effect of donor was regressed out using batch correction.
Blinding	The Investigators were not blinded to allocation during experiments and outcome assessment. However, transcriptomics data collection in a high throughput screen is not expected to be influenced by subjective factors.

Reporting for specific materials, systems and methods

We require information from authors about some types of materials, experimental systems and methods used in many studies. Here, indicate whether each material, system or method listed is relevant to your study. If you are not sure if a list item applies to your research, read the appropriate section before selecting a response.

Materials & experimental systems

n/a	Involved in the study
☒	☐ Antibodies
☒	☐ Eukaryotic cell lines
☒	☐ Palaeontology and archaeology
☒	☐ Animals and other organisms
☒	☐ Human research participants
☒	☐ Clinical data
☒	☐ Dual use research of concern

Methods

n/a	Involved in the study
☒	☐ ChIP-seq
☒	☐ Flow cytometry
☒	☐ MRI-based neuroimaging
